# Genomic and transcriptomic insights into vertebrate host-specific *Lactobacillus johnsonii* adaptation in the gastrointestinal tract

**DOI:** 10.1128/msphere.00052-25

**Published:** 2025-05-13

**Authors:** Keerthikka Ravi, Nicole R. Falkowski, Gary B. Huffnagle

**Affiliations:** 1 Department of Molecular, Cellular & Developmental Biology, University of Michigan 1259, Ann Arbor, Michigan, USA; 2 Division of Pulmonary and Critical Care Medicine, Department of Internal Medicine, University of Michigan Medical School 12266 , Ann Arbor, Michigan, USA; 3 Mary H. Weiser Food Allergy Center, University of Michigan 1259, Ann Arbor, Michigan, USA; University of California Davis, Davis, California, USA

**Keywords:** lactic acid bacteria, host-specific genes, tyrosine decarboxylase, accessory secretory pathway, strain heterogeneity, pangenome

## Abstract

We conducted a comparative genomic analysis of *Lactobacillus johnsonii* strains isolated from the gastrointestinal tract of diverse vertebrate hosts to explore the genetic basis of host specificity. We then utilized transcriptomics analysis to investigate the expression profile of identified rodent-specific genes in mouse isolate MR1 during *in vitro* and *in vivo* growth conditions. There was significant heterogeneity among strains, in both genome sequence and content, with phylogenetic clustering of strains into distinct clades associated with rodent or avian sources. There were not sufficient genomes to identify whether porcine isolates formed their own genetic clade. However, human isolates did not form a distinct clade. Functional enrichment analysis revealed significant enrichment of several genes, including surface proteins and accessory secretory pathway-related genes, as well as tyrosine decarboxylase genes in rodent isolates compared to avian isolates, including in mouse isolate MR1. A total of 40 genes were identified as rodent-associated, and all were transcriptionally active in *L. johnsonii* MR1. The global transcriptomic analysis of *L. johnsonii* MR1 was done using cells grown anaerobically, at 37˚C, under both the late-exponential phase and stationary phase, as well as during *in vivo* growth in the cecum of mono-colonized germ-free mice. Several of these genes were uniquely regulated during late exponential vs stationary phase growth and *in vivo* colonization in mice, highlighting their potential role in nutrient adaptation and host-microbe interactions.

**IMPORTANCE:**

*Lactobacillus johnsonii* is a well-known probiotic species with health-beneficial properties, including host immunomodulation and pathogen inhibition. Its growing relevance in the medical industry highlights the need to understand its biology, particularly how it adapts to different host environments. In bacteria, niche adaptation is often accompanied by the loss or gain of coding sequences along with changes in the genome size. In this study, we explored the genetic diversity of *L. johnsonii* strains from the gastrointestinal tracts of various vertebrates such as rodents, birds, swine, and humans. We found associations between genome content and host species of origin and could conceptually demonstrate that these genes are being differentially transcribed under varying conditions. Several functions were associated with specific host groups, suggesting that *L. johnsonii* strains have adapted to their hosts over time.

## INTRODUCTION

*Lactobacillus johnsonii* is a host-associated Gram-positive lactic acid bacterium (LAB). It is considered autochthonous to rodents, where strains of this species have been frequently isolated from both laboratory mice and wild rodents (1–5). In humans, *L. johnsonii* can colonize the GI tract and vagina at varying levels (6–9). Other niches frequently colonized by this species include the GI tract of swine and avian species like chickens and turkeys (10–12). *L. johnsonii* is well-studied for the health-promoting benefits it confers to the host. These include inhibition and exclusion of pathogens (13–15), immunomodulation (16, 17), and enhancement of epithelial barrier function (18) to name a few. However, the manifestations of these health benefits can vary among *L. johnsonii* strains due to strain heterogeneity, a phenomenon observed in host-adapted lactobacilli.

Host adaptation of *L. johnsonii* was first hypothesized by Guinane et al. in 2011, where through comparative analysis of 12 strains, the authors reported host-specific divergence of *L. johnsonii* strains based on genomic inversion and gene content (19). Since then, various studies, based on the genome sequence information of strains of *L. johnsonii,* have continued to build on this hypothesis. Multilocus sequence typing (MLST) and other genomic approaches have provided insights into the genetic diversity and population structure of *L. johnsonii* strains across different hosts (20). MLST-based analysis of three hypothetical genes in 47 *L*. *johnsonii* strains divided them into three main clusters, each composed entirely of isolates from avian species (chicken and turkey), humans, and mice. These results were paralleled by the findings from simple sequence repeat (SSR) loci-based analysis on the same data set (20). Recent work from (21) and (22) also suggests that based on the genome sequence, strains of *L. johnsonii* cluster phylogenetically by the host source, and this distinct clustering is also seen for closely related host species like chicken and turkey (21, 22). Furthermore, preliminary experimental evaluation demonstrated that oral gavage of broiler chickens with strains isolated from chickens resulted in better overall bird performance than when gavaged with turkey isolates (21).

In rodents, *L. johnsonii* is considered a member of the autochthonous microbiome and is thought to interact closely with other microbial species and the host. Its numbers are markedly diminished in mice after broad-spectrum antibiotic treatment, and the recovery of this species in the GI tract can be inhibited or augmented by other microbial members of the microbiome (23–26). When introduced orally into mice, *L. johnsonii* can affect mucosal immunity, systemic immunity, and intestinal function (16, 27–31). *L. johnsonii* supplementation during the weaning period was shown to be able to prevent the development of experimental atopic dermatitis in adult mice (32) and could markedly alter the developing microbiome of the neonatal offspring and decrease subsequent T helper 2 (Th2) immune responses to viral infection (28). Supplementation of *L. johnsonii* has also been shown to delay the onset of type 1 diabetes (T1D) in diabetic-prone (BBDP) rats partly by interacting and modulating the innate and adaptive immune system (29, 30).

In this study, we aim to build upon existing insights by conducting a comparative genomic analysis of *L. johnsonii* strains isolated from diverse vertebrate hosts. Through pangenome and functional enrichment analyses, we seek to elucidate the genetic basis of host specificity. Furthermore, by using transcriptomics analysis, we investigate the expression profile of the identified host-specific genes in a rodent isolate MR1, in various *in vitro* and *in vivo* growth conditions. Our findings contribute to a deeper understanding of the ecological and evolutionary dynamics of host-microbe interactions in the vertebrate GI tract.

## RESULTS

### General genomic features

**Fig 1:**
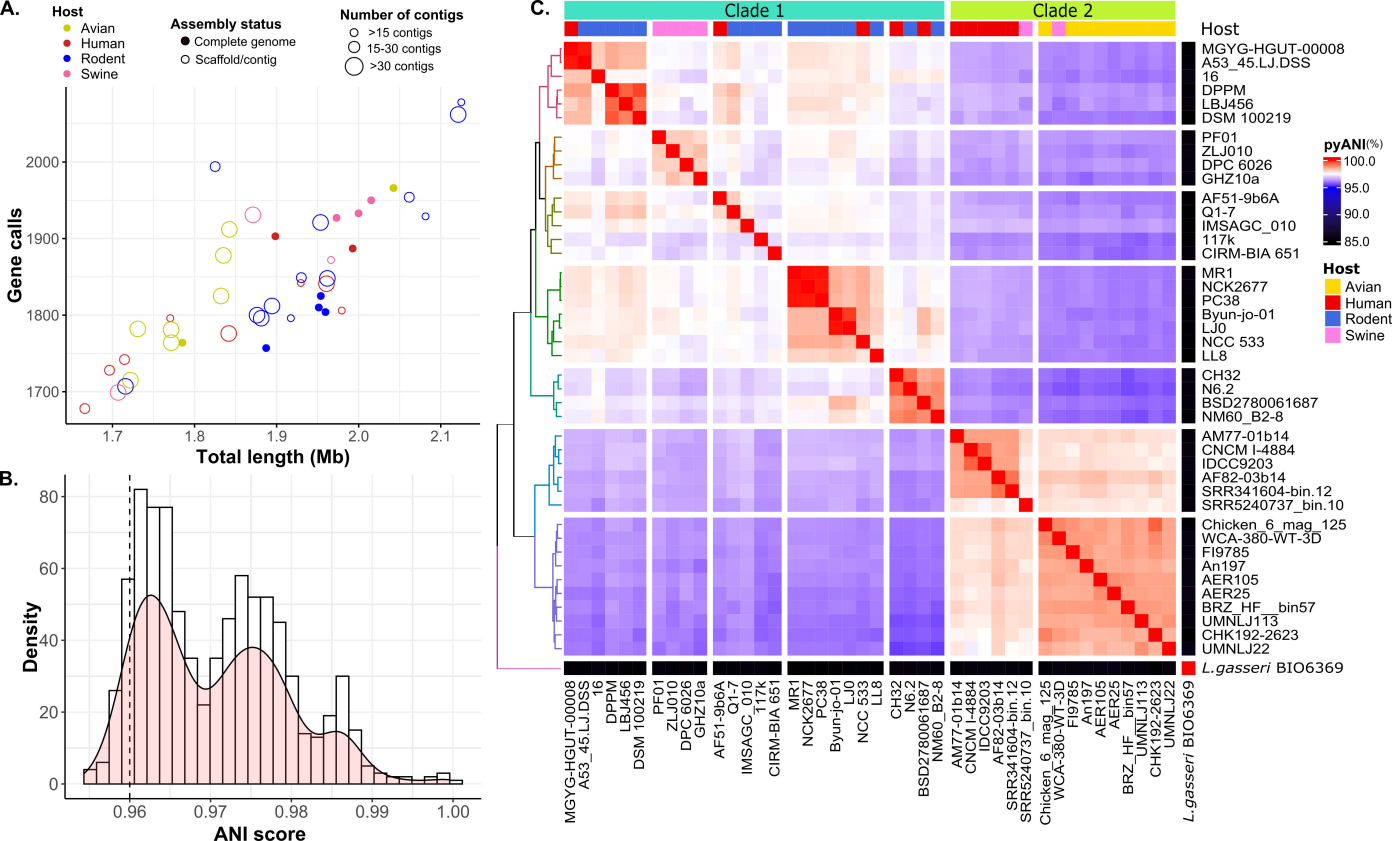
Pairwise ANI score-based clustering of *L. johnsonii* strains. (**A**) A scatter plot highlighting the genomic features of 42 vertebrate GI tract strains of *L. johnsonii*. The *x*-axis of the scatter plot represents the total genome length (Mb), and the *y*-axis represents the gene calls identified in each strain using Prodigal. Each point represents a genome, colored based on the host they originate from, as indicated in the legend. Closed and open circles represent completely assembled and draft genome sequences, respectively, and the size of the circle represents the number of contigs in the genome. (**B**) Histogram distribution of pairwise whole-genome sequence ANI scores of 42 *L. johnsonii* strains. The density plot appears to have a trimodal distribution. (**C**) Dendrogram and hierarchical clustering of whole-genome sequence ANI scores. The heat map represents the pairwise ANI scores ranging from 85% (black) to 100% (red). *L. gasseri* BIO6369 is set as the outgroup for the analysis. Ward.D2 hierarchical clustering method was used to generate a dendrogram on the Euclidean distance matrix of ANI scores. The annotation bar at the top of the heat map indicates the host that each strain originates from (Host). Based on the ANI scores, *L. johnsonii* strains cluster into two distinct clades identified at the top of the heat map: Clade 1 and Clade 2.

To create a robust data set for comparing *L. johnsonii* strains, we carefully curated genome sequences from the National Center for Biotechnology Information (NCBI) database, using specific selection criteria to ensure diverse representation and minimize bias. At the time of this study, there were 83 genome sequences of *L. johnsonii* available in the NCBI database. Of these, 55 genome sequences were from strains isolated from the GI tracts of vertebrate hosts. For a robust comparative analysis, it was essential to have a sufficient number of strains representing each host. Consequently, four strains isolated from macaque, cattle, and horse were excluded due to an insufficient number of representative strains for these hosts. Similarly, to prevent sampling bias, strains submitted under the same BioProject ID were further filtered. A representative strain was selected from such BioProject submissions, by setting the following thresholds: (i) pairwise whole-genome sequence average nucleotide identity (ANI) score of <0.999; (ii) the least number of sequence contigs in the group; and (iii) the largest relative length of longest and shortest contigs. We identified 42 genome sequences that met this criteria, and their states of assembly were as follows: 11 complete genome sequences and 31 assemblies as either scaffolds and/or contigs (Fig. 1A; Table S1). Notably, five genome sequences were derived from metagenome assemblies. Among the complete genome sequence, four isolates, GHZ10a, PF01, FI9785, and UMNLJ22, have two plasmids each. The average sequence length of an *L. johnsonii* strain in this study is 1.8 Mb, ranging from 1.6 Mb to 2.1 Mb. Open reading frames (ORFs) in the genome sequences, identified using Prodigal (33), range from 2,078 to 1,678 (Fig. 1A); of these, 1–99 gene calls were partial gene class, i.e. they either do not have a start or stop codon in the sequence. Overall, this filtering resulted in a final data set of 42 vertebrate GI tract isolates originating from humans (10 strains), rodents (17 strains), avian species including chicken (six strains) and turkey (two strains), and swine (six strains) (Table S1).

To confirm that all the strains in the data set are indeed strains of the same species, a phylogenetic relationship between the *L. johnsonii* strains was established with whole-genome ANI blast analysis (34) (Fig. 1B). A closely related species, *Lactobacillus gasseri* (BIO6369), was set as the outgroup for this analysis. The current standard for species differentiation in whole-genome ANIblast (ANIb) is <95%, i.e., if the ANI score between two genome sequences is <95%, they are considered to belong to two different species; else, they are considered as two strains of the same species (35, 36). The interspecies ANI between all *L. johnsonii* strains and *L. gasseri* was 85%, confirming the separation of all *L. johnsonii* strains and *L. gasseri* BIO6369 at the species level. The intraspecies ANI among *L. johnsonii* strains ranged from 95.4% to 99.9%, confirming that all the strains in the data set belong to the same species, *L. johnsonii*. Interestingly, the distribution of intraspecies pairwise ANI scores appeared to have a trimodal distribution with two distinct peaks at 96.4% and 97.5% similarity scores and a third smaller peak at 98.8% similarity score (Fig. 1B)**.**

To further explore the multimodal pattern in the ANI density plot, we visualized the pairwise scores as a Euclidean distance matrix, represented by the heatmap. The rows and columns of the heatmap were clustered based on the Ward.D2 method of hierarchical clustering, illustrated in Fig. 1C. The clustering of strains based on the ANI scores shows two discernible groups. When we looked at where the strains in each clade originated from, we saw that Clade 1 is composed of all the rodent isolates, and Clade 2 is composed of all the avian isolates. Human and swine isolates are scattered across these two clades.

In a supplementary analysis, we compared the genome sequences of 83 strains of *L. johnsonii* originating from various sources including dairy products. The phylogenetic tree built through whole-genome ANI analysis once again clustered the strains into two clades (Fig. S1). There was no indication of a distinct third clade, further validating the clear segregation of rodents and avian isolates. The strains isolated from various dairy products and unknown niches in vertebrate hosts were distributed among these two clades.

### Pangenome analysis of *L. johnsonii* vertebrate GI tract isolates

**Fig 2:**
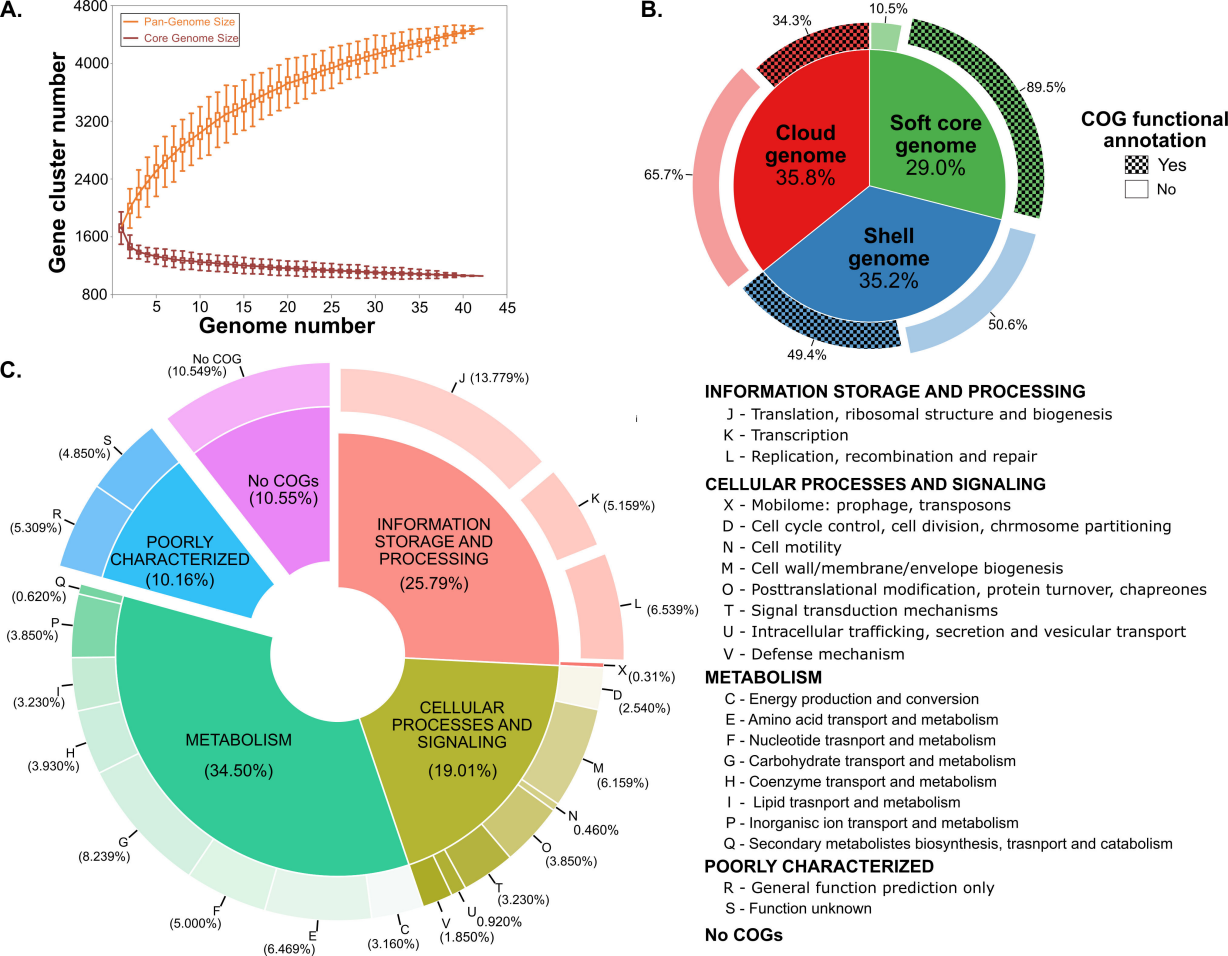
Pangenome analysis of *L. johnsonii* vertebrate GI tract isolates. (**A**) Pangenome and core genome curves indicate the number of gene clusters (GCs) added to the pangenome (orange line) and core genome (red line) with the addition of a new genome, respectively. The graph depicts that the size of the pangenome will continue to increase with the addition of more strains, while the core genome size is relatively stable. (**B**) Grouping of pangenome into three bins, soft core genome (present in ≥40 genomes), shell genome (present in 3–39 genomes), and cloud genome (present in ≤2 genomes). The inner layer represents the percentage of the pangenome in each bin. The softcore genome is composed of 1,299 GCs, while the shell genome and cloud genome have 1,577 GCs and 1,607 GCs, respectively. The other layer represents the percent of each bin that either has COG functional annotations (checkered box) or no annotations (solid box). (**C**) Distribution of COG category annotations in the softcore genome. The inner and outer circles represent the percent of the softcore genome in each COG category and subcategory, respectively. About 34% of the softcore genome is populated by GCs involved in metabolism. This includes carbohydrates, amino acids, and nucleotide transport and metabolism pathways. The second most populated category in the softcore genome is information storage and processing. This includes GCs involved in central dogma pathways, e.g., transcription, translation, and replication.

Next, we analyzed the pangenome of the 42 *L*. *johnsonii* GI tract isolates. A pangenome encompasses all the genes (ORFs) identified across all the strains in a species. Homologous genes in the pangenome are grouped into gene clusters (GCs). The pangenome consists of core and accessory genomes: GCs found in all strains of a species form the core genome, while the remaining GCs make up the accessory genome and likely represent functions involved in niche adaptation. Across the 42 *L*. *johnsonii* strains, a total of 17,440 gene calls were predicted by Prodigal (33) (Tables S1 and S2). These gene calls were clustered into 4,478 GCs using the Anvi’o microbial pangenome analysis pipeline. On average, an *L. johnsonii* strain in this study has 1,732 GCs, suggesting that the current size of the pangenome is 2.5 times the size of an average *L. johnsonii* strain. As new strains were added to the analysis, the size of the pangenome continued to increase, as shown by the pangenome profile curve, while the size of the core genome remained relatively stable (Fig. 2A).

Given that most strains in the study are in scaffolds and contigs, we refined the definitions of core and accessory genomes using the following terms and thresholds: softcore genome (GCs present in >95% of the strains), shell genome (GCs present in <95% but >5% of the strains), and cloud genome (GCs present in <5% of the strains). In this study, the softcore genome comprises 29% of the pangenome (Fig. 2B). In other words, the softcore genome (1,299 GCs) makes up 78% of an *L. johnsonii* strain in this study. The shell genome and cloud genome each represent approximately 35% of the pangenome. In terms of annotated GCs, 90% of the soft-core genome has functional annotations, from NCBI’s Clusters of Orthologous Groups (COGs) database, while the remaining 10% could not be annotated using the COG database (Fig. 2C). In contrast, only 50% and 35% of the shell and cloud genomes have functional annotations, respectively (Fig. 2B). The large sizes of the shell genome and cloud genome imply a high level of heterogeneity among these strains, and the lack of annotations of GCs in these two groups suggests that there is much that is not understood about the biological diversity of *L. johnsonii* strains.

### Association between genome sequence/content and host source of the isolate

**Fig 3:**
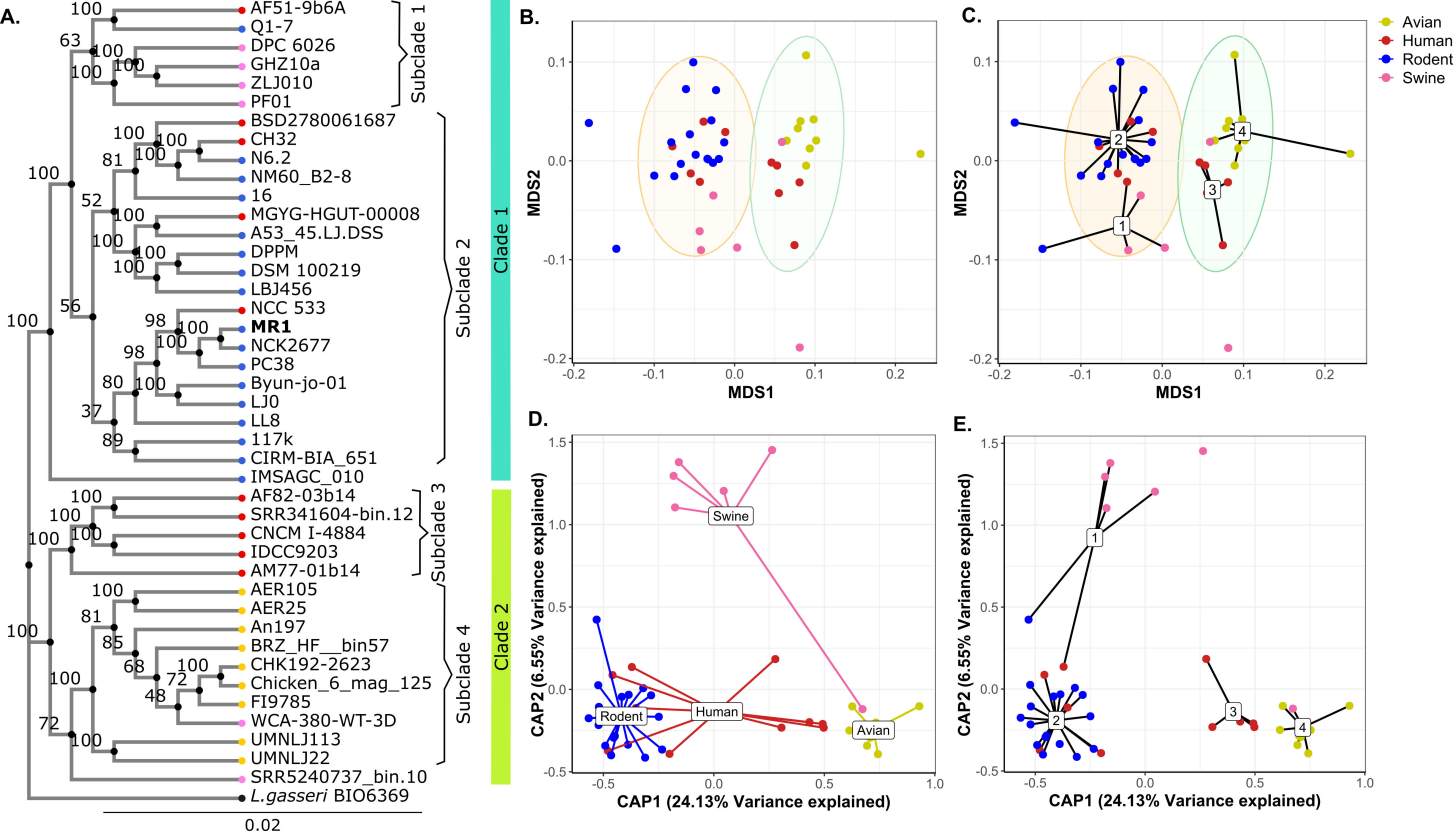
Phylogenetic association between genome sequence and strain host. (**A**) Maximum likelihood tree generated based on 378 single-copy core gene clusters across 42 *L*. *johnsonii* strains with *L. gasseri* BIO6359. The phylogenetic tree clusters the strains into two distinct clades and further into four subclades. Two strains, IMSAGC_10 and SRR5240737_bin.10, branch out early within Clade 2 and Subclade 4, respectively, and are not grouped with any of the subclades. Nodes of the tree are colored based on the host they originate from. Numbers on the tree represent the bootstrap value, and the scale bar represents the evolutionary distance. (B and C) Multidimensional scaling (MDS) ordination analysis of gene cluster presence and absence (Jaccard distance matrix). Ward.D2 hierarchical clustering of the Jaccard distance matrix separates the strains into two groups, which resemble clades 1 and 2 (**B**) and encompass the four subclades, represented by the spider plot (**C**). Ellipses denote 95% confidence intervals. (D and E) Constrained analysis of principal components (CAP) of Jaccard distance matrix, constrained by the host. The spider plots highlight the grouping of strains by hosts (**D**) and subclades (**E**). Each point represents a genome and is colored based on the host they originate from, as indicated in the legend.

To further explore our hypothesis of host specificity among *L. johnsonii* strains, we looked into the level of sequence similarity among the GCs in the core genome. Among the 1,299 GCs in the softcore genome, 367 GCs were present in all 42 strains, with each strain having only one copy of these GCs. With *L. gasseri* BIO6369 as the outgroup, a maximum likelihood (ML) tree was built using the sequence alignment of the 367 singly-copy core GCs (Fig. 3A). The ML tree generated from the alignment of these genes delineated the strains into two primary clades, mirroring the clustering observed in the ANI analysis. Within these primary clades, further resolution into four subclades was observed, which closely reflected the host source of the strains. Subclade 1 (S1) is largely populated by *L. johnsonii* strains isolated from swine, and subclade 2 (S2) predominantly comprises strains from rodents, interspersed with several human isolates. The remaining human isolates clustered into subclade 3 (S3), and all avian isolates were grouped into subclade 4 (S4). Notably, there is no correlation between the four subclades and the geographical locations (adjusted R2 of 0.05 and *P*-value 0.12) of the strains. On the other hand, the grouping of strains into the four subclades is better explained by their host source (adjusted R2 of 0.67 and *P*-value 1.481e-09), indicating that the host source may be the primary factor driving the genomic diversification of the strains observed here. In summary, this robust phylogenetic analysis corroborates the ANI-based clustering and strongly suggests that there is host-specific clustering among the GI tract vertebrate strains of *L. johnsonii*.

To further explore the association between host specificity and overall genomic content, we performed a pairwise analysis that compares the presence and absence of genes (Jaccard distance analysis) between strains and uses that as an estimate of genomic relatedness between all the strains. Based on the Jaccard distance matrix that was generated, the strains clustered into two groups, as shown by the unconstrained MDS plot (Fig. 3B and C). The strain composition of these two groups closely mirrored that of Clade 1 and Clade 2 observed in both the ML phylogenetic and ANI analyses. Furthermore, overlaying the subclades on the Jaccard distance plot reaffirmed the consistent clustering patterns, even at the subclade level. Constraining the Jaccard distance matrix analysis for the host explains 22% of the variance in the data. A principal component analysis of the host-constrained data once again grouped the strains into four clusters (Fig. 3D and E). This is consistent with the subclades identified in the ML phylogenetic analysis. In summary, we see strong evidence for host-specific clustering among *L. johnsonii* strains in this study. This host specificity is most distinct for strains originating from rodent and avian hosts. While swine isolates may demonstrate host specificity, the small sample size limits the conclusion from this data set, and there was no evidence supporting human-specific clustering of strains in this data set.

### Functional enrichment analysis

The genomic analysis strongly suggests host-specific clustering of strains, which is more pronounced for strains originating from rodent and avian isolates. To further explore this specificity, we performed a functional enrichment analysis to identify functions significantly associated with the rodent host group, using GCs annotated by the COG function database and KEGG KOfam database. Among the rodent isolates, a total of 11 COG/KOfam functions, spanning 18 GCs, were found to be significantly enriched (Table 1). Most of these functions were enriched in more than one host, either rodent and swine or rodent and human, and were rare or absent in avian hosts (Table S3). To do more in-depth computational analysis that can be complemented by *in vitro* and *in vivo* analyses, from this point onward, we focused on rodent isolate MR1 as a representative strain for the rodent strains in the study. Notably, the whole-genome sequence-based pairwise ANI score of *L. johnsonii* MR1 strain and the other rodent strains ranges from 97.3 to 99.9% (Table S4). Through pBLAST analysis, we identified 17 CDS in *L. johnsonii* MR1 strain that were homologous to the 18 rodent-enriched GCs (Table 2). Furthermore, through nucleotide BLAST analysis, we confirmed that all CDS identified were present in rodent isolates and were rare or absent in avian isolates.

**TABLE 1:** COG/KOfam functions identified as significantly enriched in rodent strains

Function enriched in rodent isolates (COG/Kofam)	Enrichment score	Adjusted *q*-value	Accession (COG/KEGG ID)	Gene clusters ID(s)
DNA-binding transcriptional regulator, IscR family (IscR) (PDB:1XD7)	32.31	5.40E−04	COG1959	GC_00001563
Multidrug resistance efflux pump EmrA (EmrA) (PDB:4TKO)	23.82	4.10E−03	COG1566	GC_00000091
ABC-type bacteriocin/lantibiotic exporters, contain an N-terminal double-glycine peptidase domain (SunT) (PDB:3K8U)	22.71	6.10E−03	COG2274	GC_00000094
Periplasmic protein TonB, links inner and outer membranes (TonB) (PDB:1IHR)!!!Uncharacterized conserved protein YjbI, contains pentapeptide repeats (YjbI) (PDB:2BM4)	20.89	1.30E−02	COG0810!!! COG1357	GC_00001573, GC_00001922, GC_00002002, GC_00003025, and GC_00003157
UDP-glucose:(galactosyl) LPS alpha-1,2-glucosyltransferase [EC:2.4.1.58]	32.82	3.60E−04	K03279	GC_00001444 and GC_00001452
HTH-type transcriptional regulator, cell division transcriptional repressor	25.75	3.60E−03	K22300	GC_00001504 and GC_00002348
Two-component system, LytTR family, sensor histidine kinase ComD [EC:2.7.13.3]	22.71	5.50E−03	K12294	GC_00001408
Accessory secretory protein Asp1	22.18	5.50E−03	K12268	GC_00001536 and GC_00002953
Accessory secretory protein Asp3	22.18	5.50E−03	K12270	GC_00001548
Poly(glycerol-phosphate) alpha-glucosyltransferase [EC:2.4.1.52]	22.18	5.50E−03	K00712	GC_00000072
Accessory secretory protein Asp2	22.18	5.50E−03	K12269	GC_00001532

**TABLE 2:** Rodent-enriched and unique genes (genome locations in *L. johnsonii* MR1)^*a*^

Gene cluster ID	Enriched/ unique	Protein ID	Locus tag	Genes	Product	Start	End	Functional group
GC_00001563	Enriched	UKV65942.1	LXB09_04170	LXB09_04170	Rrf2 family transcriptional regulator	893,030	893,395	Transcription, replication, and recombination
GC_00001504	Enriched	UKV66033.1	LXB09_04240	LXB09_04240	Helix-turn-helix domain-containing protein	903,115	904,092	Transcription, replication, and recombination
GC_00001982	Unique	UKV64487.1	LXB09_05425	LXB09_05425	RNA helicase domain-containing protein	1,163,574	1,164,146	Transcription, replication, and recombination
GC_00001996	Unique	UKV64488.1	LXB09_05430	LXB09_05430	Replication protein	1,164,101	1,164,664	Transcription, replication, and recombination
GC_00001918	Unique	UKV64492.1	LXB09_05455	LXB09_05455	Metalloregulator ArsR/SmtB family transcription factor	1,168,316	1,168,669	Transcription, replication, and recombination
GC_00002657	Unique	UKV65021.1	LXB09_08650	LXB09_08650	Helix-turn-helix domain-containing protein	1,821,892	1,822,635	Transcription, replication, and recombination
GC_00001573	Enriched	UKV65852.1	LXB09_03700	LXB09_03700	LPXTG cell wall anchor domain-containing protein	796,306	806,622	Cell wall/membrane-associated
GC_00001452	Enriched	UKV64440.1	LXB09_05160	LXB09_05160	Glycosyltransferase family 8 protein	1,094,352	1,095,302	Cell wall-/membrane-associated
GC_00001444	Enriched	UKV64441.1	LXB09_05165	LXB09_05165	Glycosyltransferase family 8 protein	1,095,315	1,096,265	Cell wall-/membrane-associated
GC_00002002	Enriched	UKV64453.1	LXB09_05230	LXB09_05230	LPXTG cell wall anchor domain-containing protein	1,112,945	1,119,547	Cell wall-/membrane-associated
GC_00002002	Enriched	UKV64529.1	LXB09_05660	LXB09_05660	YSIRK-type signal peptide-containing protein	1,224,733	1,230,180	Cell wall-/membrane-associated
GC_00002002	Enriched	UKV64702.1	LXB09_06670	LXB09_06670	YPDG domain-containing protein	1,440,040	1,447,182	Cell wall-/membrane-associated
GC_00002392	Unique	UKV65177.1	LXB09_00090	chbA	PTS lactose/cellobiose transporter subunit IIA	16,870	17,211	Metabolism
GC_00001884	Unique	UKV65178.1	LXB09_00095	bglA	Glycoside hydrolase family 1 protein	17,211	18,680	Metabolism
GC_00001408	Enriched	UKV65639.1	LXB09_02555	LXB09_02556	GHKL domain-containing protein	544,799	546,082	Metabolism
GC_00002433	Unique	UKV65672.1	LXB09_02720	tyrDC	Tyrosine decarboxylase	578,368	580,206	Metabolism
GC_00002692	Unique	UKV65673.1	LXB09_02725	tyrP	APC family permease	580,226	581,662	Metabolism
GC_00001892	Unique	UKV64493.1	LXB09_05460	LXB09_05460	CadD family cadmium resistance transporter	1,168,686	1,169,303	Metabolism
GC_00000072	Enriched	UKV65844.1	LXB09_03660	gtfB	Accessory Sec system glycosylation chaperone GtfB	783,635	784,963	Accessory secretory pathway
GC_00000072	Enriched	UKV65845.1	LXB09_03665	gtfA	Accessory Sec system glycosyltransferase GtfA	784,956	786,485	Accessory secretory pathway
GC_00001548	Enriched	UKV65847.1	LXB09_03675	asp3	Accessory Sec system protein Asp3	788,869	789,726	Accessory secretory pathway
GC_00001532	Enriched	UKV65848.1	LXB09_03680	asp2	Accessory Sec system protein Asp2	789,710	791,254	Accessory secretory pathway
GC_00001536	Enriched	UKV65849.1	LXB09_03685	asp1	Accessory Sec system protein Asp1	791,256	792,764	Accessory secretory pathway
GC_00002010	Unique	UKV65223.1	LXB09_00325	hsdS	Restriction endonuclease subunit S	70,535	71,539	Defense mechanisms
GC_00002220	Unique	UKV65226.1	LXB09_00340	hsdS	Restriction endonuclease subunit S	73,023	74,150	Defense mechanisms
GC_00000091	Enriched	UKV65629.1	LXB09_02500	blpB	Hypothetical protein	534,936	535,529	Defense mechanisms
GC_00000091	Enriched	UKV65634.1	LXB09_02530	blpB	HlyD family efflux transporter periplasmic adapter subunit	538,231	538,824	Defense mechanisms
GC_00000094	Enriched	UKV65635.1	LXB09_02535	blpA	Peptide cleavage/export ABC transporter	538,835	540,994	Defense mechanisms
GC_00001766	Unique	UKV64485.1	LXB09_05415	LXB09_05415	Phage integrase SAM-like domain-containing protein	1,161,997	1,163,181	Mobilome
GC_00002522	Unique	UKV65042.1	LXB09_08770	LXB09_08770	Phage portal protein	1,832,742	1,834,169	Mobilome
GC_00002197	Unique	UKV65043.1	LXB09_08775	LXB09_08775	Hypothetical protein	1,834,147	1,834,578	Mobilome
GC_00002378	Unique	UKV65171.1	LXB09_00060	LXB09_00061	Hypothetical protein	10,408	11,757	Unknown function
GC_00002271	Unique	UKV65181.1	LXB09_00110	LXB09_00110	Hypothetical protein	22,546	23,736	Unknown function
GC_00002188	Unique	UKV65182.1	LXB09_00115	LXB09_00115	ATP-binding protein	23,990	25,267	Unknown function
GC_00002003	Unique	UKV64491.1	LXB09_05450	LXB09_05450	Hypothetical protein	1,166,859	1,167,836	Unknown function
GC_00002752	Unique	UKV65010.1	LXB09_08595	LXB09_08595	Hypothetical protein	1,816,830	1,817,231	Unknown function
GC_00002653	Unique	UKV65011.1	LXB09_08600	LXB09_08600	PH domain-containing protein	1,817,253	1,818,152	Unknown function
GC_00002652	Unique	UKV65020.1	LXB09_08645	LXB09_08645	ERF family protein	1,821,120	1,821,878	Unknown function
GC_00002683	Unique	UKV65028.1	LXB09_08685	LXB09_08685	Hypothetical protein	1,825,077	1,825,340	Unknown function
GC_00002687	Unique	UKV65039.1	LXB09_08755	LXB09_08755	Hypothetical protein	1,830,100	1,830,648	Unknown function

^*a*^ Rodent-enriched genes were identified using anvi'o functional enrichment analysis. Rodent unique genes were identified by filtering gene clusters that were only found in the rodent isolate and also present in strain M1. Homologs of gene clusters in *L. johnsonii* MR1 were then identified using the amino acid sequence of gene clusters and pBLAST and tBLASTn analysis.

Four surface proteins of varying lengths were identified as rodent-specific genes in *L. johnsonii* MR1. The schematic representation of these genes is shown in Fig. S2. These genes encode the C-terminal anchoring motif, LPxTG, and the N-terminal YSIRK signal sequence that are found on surface proteins in gram-positive bacteria (37). Apart from LPxTG and YSIRK motifs, these genes also contain other domains that are associated with surface proteins in lactobacilli, like the Muc_B2 domain (38), Rib domain (39), and mucus adhesin domain homologous to Lar_0958 adhesin in *Limosilactobacillus reuteri* (40). Additionally, genes encoding glycosyltransferase family 8 (GT8) proteins were also among the rodent host-specific genes in *L. johnsonii*. Similar GT8 proteins are also identified in *Levilactobacillus brevis* (*gtf_D15_*) and *Lactiplantibacillus paraplantarum* BGCG11 (*orf2*), where they are found to be associated with cell envelope and eps cluster, respectively (41, 42). Other rodent-specific genes of interest include bacteriocin exporters, *blpA* and *blpB* (LXB09_02500 and LXB09_2530)*,* and accessory secretory (aSec) pathway genes *asp1/2/3,* glycosyltransferases *gtfA* and glycosyltransferase *gtfB*. The schematic representation of these genes is shown in Fig. S3.

**Fig 4:**
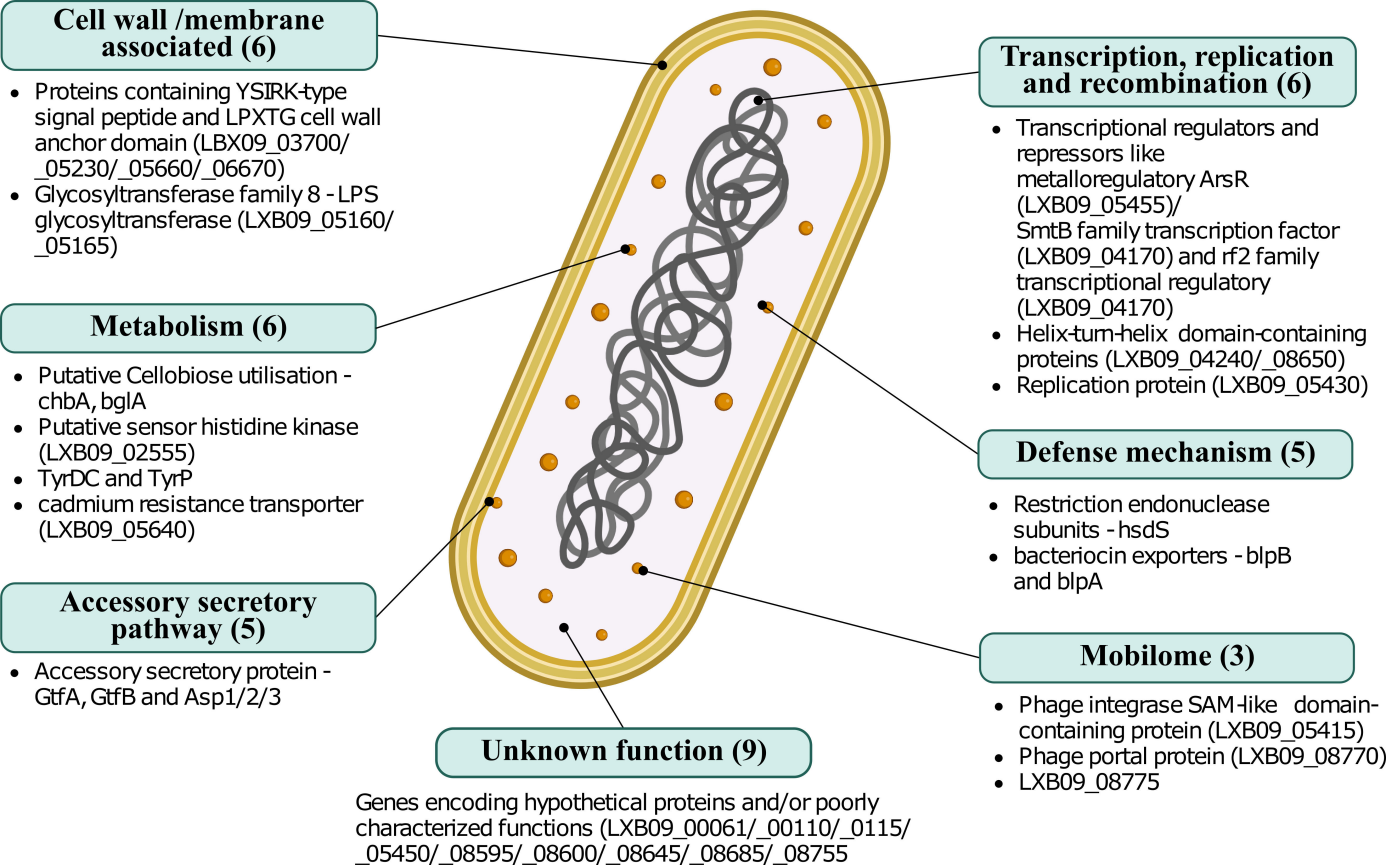
Functions in MR1 strains, identified as significantly enriched or unique to rodent isolates. Through functional analysis of the pangenome, 16 genes in *L. johnsonii* MR1 were identified as significantly enriched among rodent isolates and absent among avian isolates. Additionally, 23 genes were identified as unique to rodent isolates and found in the MR1 strain. The 39 genes span across seven functional categories and pathways like transcription, replication, recombination, and cell wall/membrane associated. Created with BioRender.com.

The functional enrichment analysis identifies COG/KOfam functions that are significantly associated with a group; however, this type of analysis excludes GCs that have no annotated functions, and 46% of the GCs in this pangenome have no functional annotations. Therefore, we also identified GCs that were unique to rodent isolates, irrespective of functional annotation. This approach identified 25 GCs unique to multiple rodent isolates, including *L. johnsonii* MR1, which were absent in strains from other hosts. Through pBLAST analysis, these 25 GCs were found to be homologous to 23 CDS in *L. johnsonii* MR1 (Table 2). Some of these genes include tyrosine decarboxylase t*yrDC* and its transporter *tyrP*, involved in converting tyrosine taken into the cell through TyrP, into tyramine and exported out through the same antiporter, PTS cellobiose transporter subunit IIA *chbA* and 6-phospho-β-glucosidase (Fig. S3). The 6-phospho-β-glucosidase, identified as *bglA* through KEGG analysis, is homologous to aryl-6-phospho-β-glucosidase *blgA* and *bglH* found in *Bacillus subtilis* and facilitates the growth of cells on aryl-β-d-glucosides like salicin and arbutin (43). Other rodent unique genes identified encode for restriction endonuclease subunit S *hsdS*, phage proteins, and several proteins of yet unknown functions. Altogether, 40 CDS were identified in *L. johnsonii* MR1 as either significantly enriched in rodent isolates or unique to some rodent isolates (Fig. 4). The presence of 40 genes, potentially involved in adhesion, nutrient utilization, niche adaptation, and defense, suggests that these functions may be driving some of the host specificity observed in the phylogenomic analysis.

### *In vitro* and *in vivo* transcriptional activity of putative rodent-specific genes in *L. johnsonii* MR1

**Fig 5:**
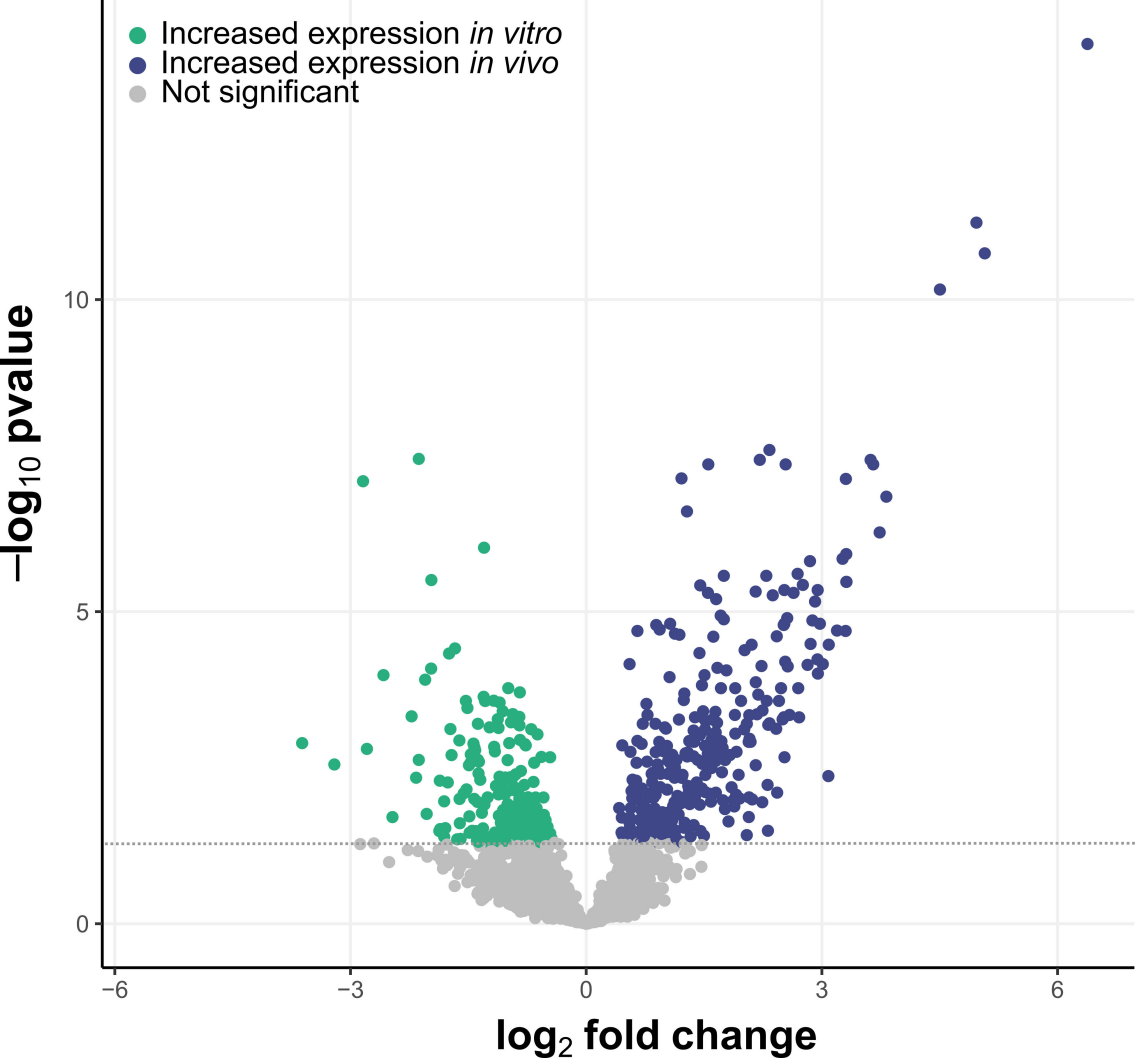
Differential expression of *L. johnsonii* MR1 genes during *in vitro* and *in vivo* growth conditions. Schematic representation of RNA-seq data (Table S5), comparing the expression of genes during the anaerobic late exponential growth phase (green dots) and during *in vivo* colonization of germ-free mice cecum (blue dots). Genes are considered to have a significant change in expression if the adjusted *P*-value is <0.05. The adjusted *P*-value threshold (=0.05) is reported by the dotted gray line.

Through computational analysis, we identified 40 genes as rodent-associated in *L. johnsonii* MR1. Next, we wanted to investigate if the predicted genes are transcriptionally active in this strain. For this, a global transcriptomic analysis of *L. johnsonii* MR1 during anaerobic *in vitro* growth and *in vivo* during host colonization was done. For the *in vitro* analysis, cells grown anaerobically at 37˚C were collected at the late-exponential phase and stationary phase. For the *in vivo* analysis, cecal contents of germ-free Balb/c mice, 7 days after oral gavage with *L. johnsonii* MR1, were collected. Stable colonization of the bacterium in the GI tract in mice was confirmed by monitoring CFU levels in the feces (Fig. S4). Global transcriptomics analysis identified 497 genes with a significant change in the expression, with 310 genes having higher expression during *in vivo* colonization and 187 genes having higher expression during the *in vitro* late-exponential growth phase (Fig. 5; Table S5).

**Fig 6:**
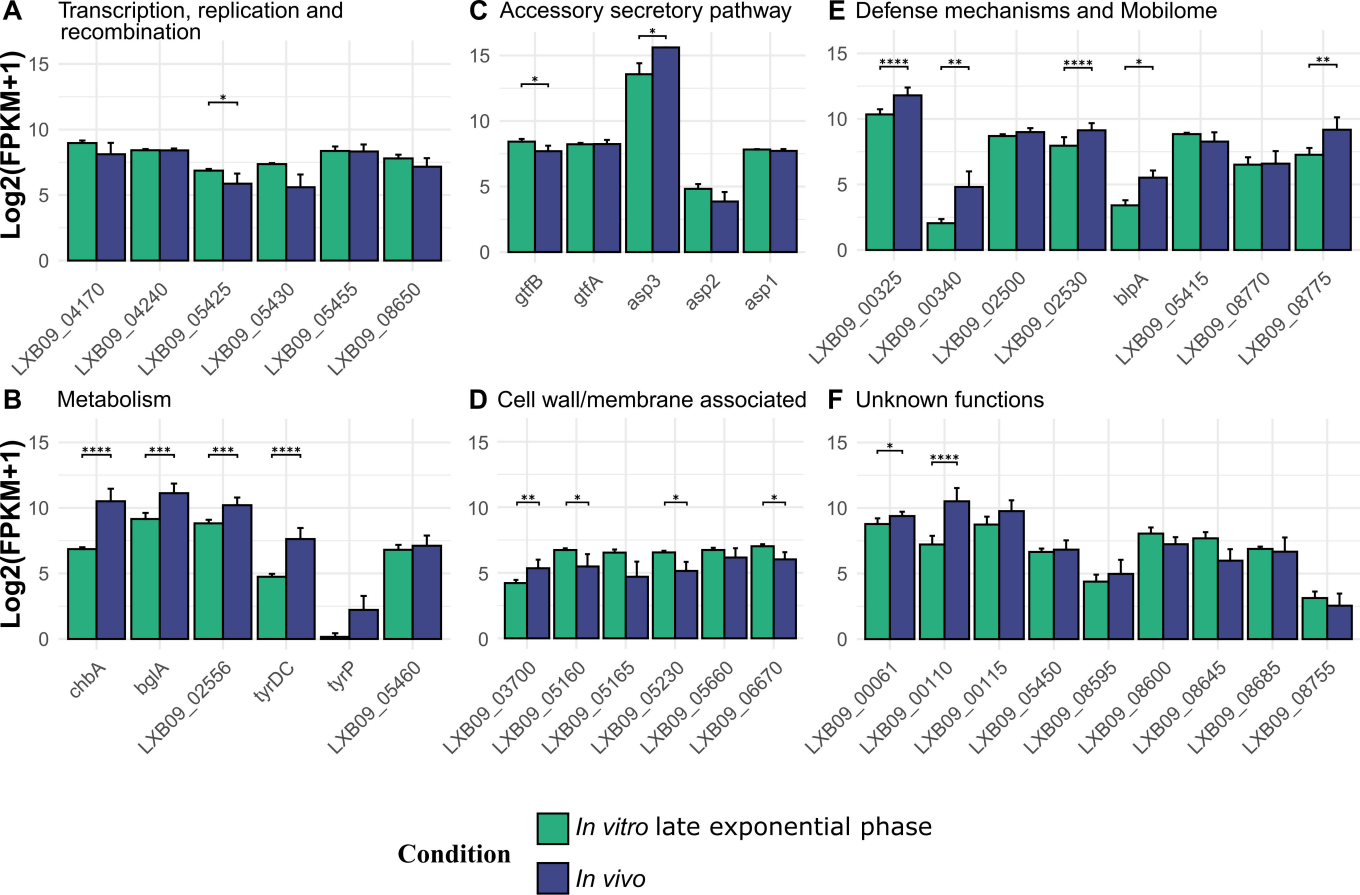
*In vitro* and *in vivo* expressions of rodent-specific genes in *L. johnsonii* MR1. Bar plot representing fragments per kilobase of transcript per million mapped (FPKM) reads of *L. johnsonii* during the anaerobic late exponential growth phase (green bars) and during *in vivo* colonization of germ-free mice cecum (blue bars). Genes are binned into groups based on their functional annotations. The asterisk located above the black line indicates genes with significant changes in the expression between the two conditions. One asterisk indicates an adjusted *P*-value smaller than 0.05 (*P* < 0.05), two asterisks indicate an adjusted *P*-value smaller than 0.01 (<0.01), and three asterisks indicate an adjusted *P*-value smaller than 0.001 (*P* < 0.001).

The identified putative host-specific genes in *L. johnsonii* MR1 were found to be transcriptionally active under both *in vitro* and *in vivo* conditions (Fig. 6; Fig. S5; Table S6). Genes belonging to the central dogma of biological processes like transcription and replication were all expressed in both conditions. Similarly, genes belonging to the aSec pathway were also expressed *in vitro* and *in vivo,* with *asp3* having a significantly higher expression *in vivo*. Most genes associated with cell wall/membrane had higher expression *in vitro* than *in vivo*, except the surface protein LXB09_03700, encoding the Muc B2-like domain and the Lar0958_adhesion superfamily domain. Interestingly, metabolism-related genes like *chbA*, *bglA,* LXB09_02555, and *tyrDC* had a significantly higher expression *in vivo*.

Other genes of interest with a significantly higher expression *in vivo* include restriction endonuclease subunit S (LXB09_00325 and LXB09_00340) and putative bacteriocin exporter (LXB09_02530 and *blpA*). In summary, all 40 genes were transcriptionally active during host colonization, with significant upregulation of the surface protein gene (LXB009_03700), acid tolerance genes (*tyrp* and *tyrDC*), and carbohydrate utilization genes (*chbA* and *bglA*) *in vivo*.

## DISCUSSION

Host adaptation of *L. johnsonii* strains was first hypothesized in 2012 (20), and since then, there has been mounting evidence suggesting that *L. johnsonii* strains cluster into phylogenetic clades represented by mammalian and avian hosts. Even among avian isolates, given a large enough data set, distinct clustering of turkey and chicken isolates is observed (21). The early branching of rodent and avian isolates observed here supports the concept that the strains in these two clades are more adapted to their hosts. This observation is consistent with the characterization of *L. johnsonii* as an autochthonous colonizer in rodents and birds. The adaptation of porcine isolates appears to be a more recent and ongoing process, and while the data set of gastrointestinal isolates was complete at the time of analysis, it comprises only a small number of porcine isolates. Thus, the autochthonous vs allochthonous nature of *L. johnsonii* colonization in pigs remains open.

In our analyses, functions such as adhesion, carbohydrate transport and metabolism, and cell protection (antibiotic resistance and antimicrobial activity) were key factors underlying the clustering of strains into distinct host-specific phylogenetic clades. More specifically, these included differences in surface proteins, glycosyl transferases, ABC transporters, restriction endonuclease subunit S, and bacteriocin transporter subunits. These observations are consistent with our understanding that GI tract physiological and anatomical parameters exhibit host inter-species differences along the length of the GI tract, including mucus thickness, pH, gastric acid secretion, and metabolites like bile acids, cholesterol, and phospholipids (44). For lactobacilli, which can colonize multiple hosts, host specificity will be influenced by their ability to adhere to gut epithelial cells, utilize the micronutrients available in the niche, produce inhibitory substances, and survive and grow in the gut environment (45). Our results provide additional support for this conceptual framework. Strain specificity seen in *L. johnsonii* has been previously reported to be associated with differences in cell surface proteins, phage components, and cell protection systems such as restriction–modification and CRISPR-Cas9 systems (19). More recent work has reported the presence of several antibiotic resistance genes in the turkey isolates of *L. johnsonii* but not in strains from other host sources (21).

*L. johnsonii*, as a member of the autochthonous microbiome, will interact closely with other microbial species and the host. Existing literature suggests that *L. johnsonii* cohabitates the same niche as the only other dominant lactobacilli in rodents: *L. reuteri*, and *L. johnsonii* may have closely evolved from *L. reuteri (46, 47*). Similar to *L. johnsonii,* there is extensive evidence of host-specific phylogenetic clustering in *L. reuteri* strains isolated from rodents, avians, swine, and humans (48–51). Experimental studies further underscore the exceptional host adaptation of *L. reuteri* to rodents and chickens (48). In one study, *L. johnsonii* was shown to form a mixed-species biofilm with *L. reuteri,* in the forestomach (52). However, it has been hypothesized that the cohabitation of the same niche by *L. johnsonii* and *L. reuteri* is facilitated by nutritional adaptation, where during cohabitation, *L. reuteri* utilizes maltose, instead of glucose, making glucose available to *L. johnsonii* (53).

Here, we identified that genes in the aSec pathway are rodent-specific. In gram-positive bacteria, the aSec pathway consists of seven conserved proteins, SecA2, SecY2, Asp1/2/3, GtfA, and GtfB, which facilitate glycosylation and secretion of serine repeat-rich proteins (SRRPs) (54–56). In *L. johnsonii*, two aSec pathway genes, *secA2* and *secY2*, have been previously reported to be host-specific genes in rodents (22). *L. johnsonii* MR1 also encodes an SRRP gene; however, this is not well-characterized. In *L. reuteri,* surface-exposed SRRP (Lr70902) has been shown to facilitate biofilm formation during colonization of the forestomach (57). In *L. reuteri*, *secA2* and Lr70902 were identified as rodent host-specific (51, 58). Loss of either gene in *L. reuteri* decreased the fitness of the strain during host GI tract colonization (57). Notably, several genes identified as rodent-specific in *L. johnsonii*—such as those encoding the aSec pathway—are similarly rodent-specific in *L. reuteri*, highlighting similar convergent adaptation mechanisms employed by the two species to their shared host environment.

We identified four surface proteins as rodent host-specific in *L. johnsonii* MR1. The LXB09_03700 surface protein was found downstream of aSec pathway genes, typically where the SRRP secreted by the pathway is found. The identified surface proteins encoded several adhesion-related domains like the MUB domain. The MUB domain, found exclusively in lactobacilli, plays a crucial role in their adherence to the mucus layer in the lower gastrointestinal tract. The number of MUB domains present in a single protein often varies from 1 to 15, and this difference is also seen among orthologous proteins in closely related species (59). Notably, MUB-like proteins also show a high genetic heterogeneity among strains (60), similar to what was observed here in the *L. johnsonii* MR1 strain. Another repetitive domain observed in rodent-associated surface proteins in *L. johnsonii* MR1 is the lar0958_adhesion superfamily and the Rib domain. Lar_0958 is a host-specific surface protein found in *L. reuteri* human isolate, the loss of which significantly impacted mucus adhesion properties of the cell (40). Similarly, rib domains are found in a diverse range of surface proteins in gram-positive bacteria including *L. reuteri* and *L. acidophilus* (61, 62). From previous work done in our lab, we know that *L. johnsonii* can colonize both the upper and lower GI tracts in rodents. It will be interesting to see if these host-specific surface proteins, encoding various adhesion domains, play a role in establishing mixed-species biofilm in the upper GI tract and facilitating colonization in the mucus layer in the lower GI tract.

Finally, another interesting rodent unique function that we identified here is tyrosine decarboxylation. Decarboxylase pathways (arginine, glutamate, tyrosine, and histamine), as shown in *L. reuteri, Levilactobacillus brevis, Enterococcus faecium,* and *Limosilactobacillus vaginalis,* often play a dual role in supplying energy to the cells and participating in pH regulation by generating a proton motive force (63–68). In animals, pH levels typically vary across the length of the GI tract, with the lowest levels being in the stomach/gizzard (44, 69). In rodents, the pH across the small intestine to the colon is 4–5.5. However, in humans and swine, this range is much higher, pH 6–7. Similarly, in chickens, the pH in the small intestine and colon ranges within 5–8. The difference in pH levels in the GI tract among animals could be a contributing factor to the selection of specific acid tolerance mechanisms in some strains and not others. Apart from the decarboxylase pathway, *L. reuteri* rodent isolates also encode urease activity for acid tolerance (51). While like other lactic acid bacteria, *L. johnsonii* may have more than one acid tolerance mechanism, the conservation of tyrosine decarboxylase genes in rodent isolates suggests that they may be a unique fitness factor for colonizing the low pH niches in the rodent GI tract.

Our analysis is consistent with the hypothesis that *L. johnsonii* is an allochthonous colonizer in the human GI tract. While *L. johnsonii* is detected in the GI tract in humans after administration, this colonization is transient and often strain-dependent (70, 71). The human isolates in this study demonstrate two distinct trends in the phylogenetic analysis. While 5 out of 10 strains colocalize with rodent and swine isolates, the remaining five isolates cluster together, forming a distinct subclade, S3. The close association of human isolates with either rodents or avian strains is also observed in *L. reuteri* strains, where it is hypothesized to be due to zoonotic transmission in humans from rodents and domesticated poultry (49). If the *L. johnsonii* human isolates were originally acquired through zoonotic transmission, then these strains should also be able to colonize other hosts like rodents or poultry/chicken, and preliminary analysis suggests that this is the case. The human isolate NCC 533, clustering with *L. johnsonii* MR1 and other rodent isolates on S2, has been shown to persistently colonize, both germ-free and conventional mice (72, 73). However, the human isolate IDCC9203, found in subclade S3, is a poor colonizer of mice GI tract (74). ATCC 33200^T^ (type strain), a human blood isolate of *L. johnsonii* that is phylogenetically related to *L. johnsonii* IDCC9203 and distant from *L. johnsonii* NCC533, is also a poor colonizer of the mouse GI tract (22, 72). While more cross-host colonization experiments need to be done, it is clear that the host specificity demonstrated through computational phylogenetic analysis is also reflected in the strain’s ability to colonize a host.

## MATERIALS AND METHODS

### Comparative genomics analysis

The comparative analysis of 42 *L*. *johnsonii* strains was done according to the Anvi’o microbial pangenome analysis pipeline. The code used for the comparative genomics data analysis is available at https://github.com/krthkkrv/Genomic-and-transcriptomic-analysis-of-vertebrate-host-specific-lactobacillus-johnsonii. Briefly, genome sequences were converted to contig databases. Open reading frames (ORFs) for each database were identified using Prodigal (33). Functional annotations for identified gene calls were assigned using the NCBI COGS database and the KEGG KOfam database (75, 76). A whole genome-based average nucleotide identity analysis was then run using pyANI (34) and visualized using ComplexHeatmap in R. The pangenome of all 42 *L*. *johnsonii* genomes was computed using “anvi-pan-genome.” The completeness of the pangenome was estimated using PanGP (77) and micropan (78). Using both the COG and KEGG annotation, clade-speciﬁc, subclade, and host-specific functions were identiﬁed by the “anvi-compute-functional-enrichment-in-pan (79).” Refer to the supplemental material for the detailed protocol.

### *In vitro* growth of *L. johnsonii* MR1

*L. johnsonii* MR1 was first streaked on a fresh De Man–Rogosa–Sharpe (MRS) agar (Fisher Scientific, BD 288210) plate from glycerol stock and incubated at 37°C overnight anaerobically. A single colony from this plate was inoculated in a reduced MRS broth (Fisher Scientific, BD 288130) and incubated overnight anaerobically at 37°C. Reduced MRS broth was then inoculated with overnight culture to start with an initial OD 600 of 0.05. Cells for the late-exponential phase sample were collected after 5 hours of growth, with an OD 600 of 2.28. Cells for stationary-phase samples were collected after 10 hours of growth, with an OD 600 of 4.80. All samples were grown anaerobically, at 37°C under static conditions. Cultures were set up in quadruplicate (*n* = 4). Once collected, cells were treated with RNAprotect (Qiagen, 76506) at a 2:1 ratio and stored at −80°C until RNA isolation.

### Gnotobiotic animal colonization experiment

All germ-free mouse experiments were handled at the germ-free animal facility at the University of Michigan. Eight- to ten-week-old, female (*n* = 6) and male (*n* = 2) germ-free BALB/c mice underwent gastric colonization through oral gavage of 3 × 10^8^ CFU of *L. johnsonii* MR1. At 7 days post-colonization, the stomach, small intestine proximal and distal ends, cecal content, cecum, colon, and feces were collected for CFU plating and cecal contents for RNA isolation.

### RNA extraction and RNA sequencing

*In vitro* culture samples were first thawed and then incubated with 200 mL of lysozyme (ThermoFisher, 89833) at 15 mg/mL concentration in Tris-EDTA (TE) buffer, for 30 minutes at 37˚C. After cell lysis, RNA isolation was done using Qiagen’s RNeasy minikit. RNA isolation from the cecal contents of colonized mice was performed using phenol-chloroform extraction, followed by cleanup using the RNeasy mini kit, as previously described (80). Ribosomal depletion and library preparation were done as previously described (81). All samples were sequenced using NovoSeq, paired-end sequencing, with a sequencing depth of 33M reads per sample for *in vitro* samples and 150M reads per sample for *in vivo* samples.

### RNA-sequencing data analysis

The sequencing quality of RNA reads was first assessed using FastQC, version 0.11.8. TrimGalore, version 0.6.7, was used to trim out adapter reads, as well as remove reads less than 90 bp long. The reads were aligned to the *L. johnsonii* MR1 using Bowtie2 version 2.3.4.3 with default parameters for an end-to-end alignment. The resulting SAM files were converted to BAM files using SAMtools version 1.7. The count matrix was generated using HtSeq, version 0.12.4, and counts were imported to R for differential gene expression analysis. FPKM reads were generated, and differentially expressed genes were identified using DESeq2. Genes were identified as differentially expressed if their adjusted *P* value (Padj) was less than 0.05 at a log2 fold change of 0.

## Data Availability

*In vitro* and *in vivo* RNA sequencing data reported in this paper are available at the NCBI GEO archive (GSE271801).

## SUPPLEMENTAL MATERIAL

Supplemental information msphere.00052-25-s0001.docx Supplemental text, figures, and table captions.

Table S1 msphere.00052-25-s0002.xlsx *L. johnsonii* strains used in the comparative genomic analysis.

Table S2 msphere.00052-25-s0003.xlsx Pangenome summary of 42 *L. johnsonii* vertebrate GI tract isolates.

Table S3 msphere.00052-25-s0004.xlsx Functional enrichment analysis across strains in four host groups.

Table S4 msphere.00052-25-s0005.xlsx Pairwise whole genome sequence average nucleotide identity score for 42 *L. johnsonii* strains and the outgroup *L. gasseri* BIO6369.

Table S5 msphere.00052-25-s0006.xlsx Log fold change in expression of genes in *L. johnsonii* MR1 during *in vitro* and *in vivo* growth.

Table S6 msphere.00052-25-s0007.xlsx Log fold change in expression of the 40 rodent-associated genes in *L. johnsonii* MR1 during *in vitro* and *in vivo* growth.

ASM does not own the copyrights to Supplemental Material that may be linked to, or accessed through, an article. The authors have granted ASM a non-exclusive, world-wide license to publish the Supplemental Material files. Please contact the corresponding author directly for reuse.

## References

[1] Ravi K, Rauch M, Lynch SV, Lukacs NW, Huffnagle GB. 2023. Complete genome sequence of Lactobacillus johnsonii MR1, Isolated from a BALB/c mouse cecum. Microbiol Resour Announc 12:e01078–22. doi:10.1128/mra.01078-22 36511660 PMC9872695

[2] Wu X, Zhao C, Guo Z, Hao Y, Li J, Shi H, Sun Y. 2016. Genome sequence of Lactobacillus johnsonii Strain W1, isolated from mice. Genome Announc 4:e00561. doi:10.1128/genomeA.00561-16 27313302 PMC4911481

[3] O’Flaherty S, Foley MH, Rivera AJ, Theriot CM, Barrangou R. 2020. Complete genome sequence of Lactobacillus johnsonii NCK2677, isolated from mice. Microbiol Resour Announc 9:e00918-20. doi:10.1128/MRA.00918-20 33093042 PMC7585840

[4] Leonard MT, Valladares RB, Ardissone A, Gonzalez CF, Lorca GL, Triplett EW. 2014. Complete genome sequences of Lactobacillus johnsonii strain N6.2 and Lactobacillus reuteri strain TD1. Genome Announc 2:e00397–14. doi:10.1128/genomeA.00397-14 24812223 PMC4014691

[5] Milovic A, Bassam K, Shao H, Chatzistamou I, Tufts DM, Diuk-Wasser M, Barbour AG. 2020. Lactobacilli and other gastrointestinal microbiota of Peromyscus leucopus, reservoir host for agents of Lyme disease and other zoonoses in North America. PLoS One 15:e0231801. doi:10.1371/journal.pone.0231801 32817657 PMC7446861

[6] Guerrero-Preston R, White JR, Godoy-Vitorino F, Rodríguez-Hilario A, Navarro K, González H, Michailidi C, Jedlicka A, Canapp S, Bondy J, Dziedzic A, Mora-Lagos B, Rivera-Alvarez G, Ili-Gangas C, Brebi-Mieville P, Westra W, Koch W, Kang H, Marchionni L, Kim Y, Sidransky D. 2017. High-resolution microbiome profiling uncovers Fusobacterium nucleatum, Lactobacillus gasseri/johnsonii, and Lactobacillus vaginalis associated to oral and oropharyngeal cancer in saliva from HPV positive and HPV negative patients treated with surgery and chemo-radiation. Oncotarget 8:110931–110948. doi:10.18632/oncotarget.20677 29340028 PMC5762296

[7] Pridmore RD, Berger B, Desiere F, Vilanova D, Barretto C, Pittet AC, Zwahlen MC, Rouvet M, Altermann E, Barrangou R, Mollet B, Mercenier A, Klaenhammer T, Arigoni F, Schell MA. 2004. The genome sequence of the probiotic intestinal bacterium Lactobacillus johnsonii NCC 533. Proc Natl Acad Sci USA 101:2512–2517. doi:10.1073/pnas.0307327101 14983040 PMC356981

[8] Ahire JJ, Sahoo S, Kashikar MS, Heerekar A, Lakshmi SG, Madempudi RS. 2023. In vitro assessment of Lactobacillus crispatus UBLCp01, Lactobacillus gasseri UBLG36, and Lactobacillus johnsonii UBLJ01 as a potential vaginal probiotic candidate. Probiotics & Antimicro Prot 15:275–286. doi:10.1007/s12602-021-09838-9 34417721

[9] Boucard A-S, Florent I, Polack B, Langella P, Bermúdez-Humarán LG. 2022. Genome sequence and assessment of safety and potential probiotic traits of Lactobacillus johnsonii CNCM I-4884. Microorganisms 10:273. doi:10.3390/microorganisms10020273 35208728 PMC8876136

[10] Zhang W, Wang J, Zhang D, Liu H, Wang S, Wang Y, Ji H. 2019. Complete genome sequencing and comparative genome characterization of Lactobacillus johnsonii ZLJ010, a potential probiotic with health-promoting properties. Front Genet 10:812. doi:10.3389/fgene.2019.00812 31552103 PMC6746964

[11] Reed A, Olszewska MA, Mann A, Novoa Rama E, Thippareddi H, Singh M, den Bakker HC. 2022. Draft genome sequences of two Lactobacillus johnsonii and three Ligilactobacillus salivarius strains isolated from intestinal microbiomes of chickens. Microbiol Resour Announc 11:e0092521. doi:10.1128/mra.00925-21 35112897 PMC8812308

[12] Dec M, Nowaczek A, Stępień-Pyśniak D, Wawrzykowski J, Urban-Chmiel R. 2018. Identification and antibiotic susceptibility of lactobacilli isolated from turkeys. BMC Microbiol 18:168. doi:10.1186/s12866-018-1269-6 30373569 PMC6206647

[13] He T, Zhu YH, Yu J, Xia B, Liu X, Yang GY, Su JH, Guo L, Wang ML, Wang JF. 2019. Lactobacillus johnsonii L531 reduces pathogen load and helps maintain short-chain fatty acid levels in the intestines of pigs challenged with Salmonella enterica infantis. Vet Microbiol 230:187–194. doi:10.1016/j.vetmic.2019.02.003 30827387

[14] Charlet R, Bortolus C, Sendid B, Jawhara S. 2020. Bacteroides thetaiotaomicron and Lactobacillus johnsonii modulate intestinal inflammation and eliminate fungi via enzymatic hydrolysis of the fungal cell wall. Sci Rep 10:11510. doi:10.1038/s41598-020-68214-9 32661259 PMC7359362

[15] La Ragione RM, Narbad A, Gasson MJ, Woodward MJ. 2004. In vivo characterization of Lactobacillus johnsonii FI9785 for use as a defined competitive exclusion agent against bacterial pathogens in poultry. Lett Appl Microbiol 38:197–205. doi:10.1111/j.1472-765x.2004.01474.x 14962040

[16] Fonseca W, Lucey K, Jang S, Fujimura KE, Rasky A, Ting HA, Petersen J, Johnson CC, Boushey HA, Zoratti E, Ownby DR, Levine AM, Bobbit KR, Lynch SV, Lukacs NW. 2017. Lactobacillus johnsonii supplementation attenuates respiratory viral infection via metabolic reprogramming and immune cell modulation. Mucosal Immunol 10:1569–1580. doi:10.1038/mi.2017.13 28295020 PMC5599307

[17] Kingma SDK, Li N, Sun F, Valladares RB, Neu J, Lorca GL. 2011. Lactobacillus johnsonii N6.2 stimulates the innate immune response through toll-like receptor 9 in Caco-2 cells and increases intestinal crypt paneth cell number in biobreeding diabetes-prone rats. J Nutr 141:1023–1028. doi:10.3945/jn.110.135517 21490291

[18] Zou YJ, Xu JJ, Wang X, Zhu YH, Wu Q, Wang JF. 2020. Lactobacillus johnsonii L531 ameliorates Escherichia coli-induced cell damage via inhibiting NLRP3 inflammasome activity and promoting ATG5/ATG16L1-mediated autophagy in porcine mammary epithelial cells. Vet Sci 7:112. doi:10.3390/vetsci7030112 32823867 PMC7558184

[19] Guinane CM, Kent RM, Norberg S, Hill C, Fitzgerald GF, Stanton C, Ross RP. 2011. Host specific diversity in Lactobacillus johnsonii as evidenced by a major chromosomal inversion and phage resistance mechanisms. PLoS One 6:e18740. doi:10.1371/journal.pone.0018740 21533100 PMC3080392

[20] Buhnik-Rosenblau K, Matsko-Efimov V, Jung M, Shin H, Danin-Poleg Y, Kashi Y. 2012. Indication for Co-evolution of Lactobacillus johnsonii with its hosts. BMC Microbiol 12:149. doi:10.1186/1471-2180-12-149 22827843 PMC3503616

[21] Johnson A, Miller EA, Weber B, Figueroa CF, Aguayo JM, Johny AK, Noll S, Brannon J, Kozlowicz B, Johnson TJ. 2023. Evidence of host specificity in Lactobacillus johnsonii genomes and its influence on probiotic potential in poultry. Poult Sci 102:102858. doi:10.1016/j.psj.2023.102858 37390550 PMC10331464

[22] Chen K, Zhou X, Zhao J, Ross RP, Stanton C, Chen W, Yang B. 2023. Comparative genomics of Lactobacillus johnsonii reveals extensive intraspecific genetic variation. Food Biosci 56:103190. doi:10.1016/j.fbio.2023.103190

[23] Stark KG, Falkowski NR, Brown CA, McDonald RA, Huffnagle GB. 2022. Contribution of the microbiome, environment, and genetics to mucosal type 2 immunity and anaphylaxis in a murine food allergy model. Front Allergy 3:851993. doi:10.3389/falgy.2022.851993 35769569 PMC9234882

[24] Mason KL, Erb Downward JR, Mason KD, Falkowski NR, Eaton KA, Kao JY, Young VB, Huffnagle GB. 2012. Candida albicans and bacterial microbiota interactions in the cecum during recolonization following broad-spectrum antibiotic therapy. Infect Immun 80:3371–3380. doi:10.1128/IAI.00449-12 22778094 PMC3457555

[25] Mason KL, Erb Downward JR, Falkowski NR, Young VB, Kao JY, Huffnagle GB. 2012. Interplay between the gastric bacterial microbiota and Candida albicans during postantibiotic recolonization and gastritis. Infect Immun 80:150–158. doi:10.1128/IAI.05162-11 21986629 PMC3255670

[26] Reeves AE, Theriot CM, Bergin IL, Huffnagle GB, Schloss PD, Young VB. 2011. The interplay between microbiome dynamics and pathogen dynamics in a murine model of Clostridium difficile infection. Gut Microbes 2:145–158. doi:10.4161/gmic.2.3.16333 21804357 PMC3225775

[27] Fujimura KE, Demoor T, Rauch M, Faruqi AA, Jang S, Johnson CC, Boushey HA, Zoratti E, Ownby D, Lukacs NW, Lynch SV. 2014. House dust exposure mediates gut microbiome Lactobacillus enrichment and airway immune defense against allergens and virus infection. Proc Natl Acad Sci USA 111:805–810. doi:10.1073/pnas.1310750111 24344318 PMC3896155

[28] Fonseca W, Malinczak CA, Fujimura K, Li D, McCauley K, Li J, Best SKK, Zhu D, Rasky AJ, Johnson CC, Bermick J, Zoratti EM, Ownby D, Lynch SV, Lukacs NW, Ptaschinski C. 2021. Maternal gut microbiome regulates immunity to RSV infection in offspring. J Exp Med 218:e20210235. doi:10.1084/jem.20210235 34613328 PMC8500238

[29] Valladares R, Sankar D, Li N, Williams E, Lai K-K, Abdelgeliel AS, Gonzalez CF, Wasserfall CH, Larkin J, Schatz D, Atkinson MA, Triplett EW, Neu J, Lorca GL. 2010. Lactobacillus johnsonii N6.2 mitigates the development of type 1 diabetes in BB-DP rats. PLoS One 5:e10507. doi:10.1371/journal.pone.0010507 20463897 PMC2865539

[30] Lau K, Benitez P, Ardissone A, Wilson TD, Collins EL, Lorca G, Li N, Sankar D, Wasserfall C, Neu J, Atkinson MA, Shatz D, Triplett EW, Larkin J III. 2011. Inhibition of type 1 diabetes correlated to a Lactobacillus johnsonii N6.2-mediated Th17 bias. J Immunol 186:3538–3546. doi:10.4049/jimmunol.1001864 21317395

[31] Marcial GE, Ford AL, Haller MJ, Gezan SA, Harrison NA, Cai D, Meyer JL, Perry DJ, Atkinson MA, Wasserfall CH, Garrett T, Gonzalez CF, Brusko TM, Dahl WJ, Lorca GL. 2017. Lactobacillus johnsonii N6.2 modulates the host immune responses: a double-blind, randomized trial in healthy adults. Front Immunol 8:655. doi:10.3389/fimmu.2017.00655 28659913 PMC5466969

[32] Inoue R, Nishio A, Fukushima Y, Ushida K. 2007. Oral treatment with probiotic Lactobacillus johnsonii NCC533 (La1) for a specific part of the weaning period prevents the development of atopic dermatitis induced after maturation in model mice, NC/Nga. Br J Dermatol 156:499–509. doi:10.1111/j.1365-2133.2006.07695.x 17300240

[33] Hyatt D, Chen GL, Locascio PF, Land ML, Larimer FW, Hauser LJ. 2010. Prodigal: prokaryotic gene recognition and translation initiation site identification. BMC Bioinformatics 11:119. doi:10.1186/1471-2105-11-119 20211023 PMC2848648

[34] Pritchard L, Glover RH, Humphris S, Elphinstone JG, Toth IK. 2016. Genomics and taxonomy in diagnostics for food security: soft-rotting enterobacterial plant pathogens. Anal Methods 8:12–24. doi:10.1039/C5AY02550H

[35] Richter M, Rosselló-Móra R. 2009. Shifting the genomic gold standard for the prokaryotic species definition. Proc Natl Acad Sci USA 106:19126–19131. doi:10.1073/pnas.0906412106 19855009 PMC2776425

[36] Li F, Cheng CC, Zheng J, Liu J, Quevedo RM, Li J, Roos S, Gänzle MG, Walter J. 2021. Limosilactobacillus balticus sp. nov., Limosilactobacillus agrestis sp. nov., Limosilactobacillus albertensis sp. nov., Limosilactobacillus rudii sp. nov. and Limosilactobacillus fastidiosus sp. nov., five novel Limosilactobacillus species isolated from the vertebrate gastrointestinal tract, and proposal of six subspecies of Limosilactobacillus reuteri adapted to the gastrointestinal tract of specific vertebrate hosts. Int J Syst Evol Microbiol 71:004644. doi:10.1099/ijsem.0.004644 33533708 PMC8346765

[37] Call EK, Klaenhammer TR. 2013. Relevance and application of sortase and sortase-dependent proteins in lactic acid bacteria. Front Microbiol 4:73. doi:10.3389/fmicb.2013.00073 23579319 PMC3619620

[38] MacKenzie DA, Tailford LE, Hemmings AM, Juge N. 2009. Crystal structure of a mucus-binding protein repeat reveals an unexpected functional immunoglobulin binding activity. J Biol Chem 284:32444–32453. doi:10.1074/jbc.M109.040907 19758995 PMC2781659

[39] Wästfelt M, Stâlhammar-Carlemalm M, Delisse AM, Cabezon T, Lindahl G. 1996. Identification of a family of streptococcal surface proteins with extremely repetitive structure. J Biol Chem 271:18892–18897. doi:10.1074/jbc.271.31.18892 8702550

[40] Etzold S, MacKenzie DA, Jeffers F, Walshaw J, Roos S, Hemmings AM, Juge N. 2014. Structural and molecular insights into novel surface-exposed mucus adhesins from Lactobacillus reuteri human strains. Mol Microbiol 92:543–556. doi:10.1111/mmi.12574 24593252

[41] Zivkovic M, Miljkovic M, Ruas-Madiedo P, Strahinic I, Tolinacki M, Golic N, Kojic M. 2015. Exopolysaccharide production and ropy phenotype are determined by two gene clusters in putative probiotic strain Lactobacillus paraplantarum BGCG11. Appl Environ Microbiol 81:1387–1396. doi:10.1128/AEM.03028-14 25527533 PMC4309721

[42] Feyereisen M, Mahony J, O’Sullivan T, Boer V, van Sinderen D. 2020. A plasmid-encoded putative glycosyltransferase is involved in hop tolerance and beer spoilage in Lactobacillus brevis. Appl Environ Microbiol 86:e02268-19. doi:10.1128/AEM.02268-19 31757821 PMC6974642

[43] Setlow B, Cabrera-Hernandez A, Cabrera-Martinez RM, Setlow P. 2004. Identification of aryl-phospho-beta-D-glucosidases in Bacillus subtilis. Arch Microbiol 181:60–67. doi:10.1007/s00203-003-0628-2 14652714

[44] Hatton GB, Yadav V, Basit AW, Merchant HA. 2015. Animal farm: considerations in animal gastrointestinal physiology and relevance to drug delivery in humans. J Pharm Sci 104:2747–2776. doi:10.1002/jps.24365 25712759

[45] Nemcova R. 1997. Selection criteria of lactobacilli for probiotic use. Vet Med-Czech 42:19–27. 9123779

[46] Brooks SPJ, McAllister M, Sandoz M, Kalmokoff ML. 2003. Culture-independent phylogenetic analysis of the faecal flora of the rat. Can J Microbiol 49:589–601. doi:10.1139/w03-075 14663493

[47] Salzman NH, de Jong H, Paterson Y, Harmsen HJM, Welling GW, Bos NA. 2002. Analysis of 16S libraries of mouse gastrointestinal microflora reveals a large new group of mouse intestinal bacteria. Microbiology (Reading) 148:3651–3660. doi:10.1099/00221287-148-11-3651 12427955

[48] Duar RM, Frese SA, Lin XXB, Fernando SC, Burkey TE, Tasseva G, Peterson DA, Blom J, Wenzel CQ, Szymanski CM, Walter J. 2017. Experimental evaluation of host adaptation of Lactobacillus reuteri to different vertebrate species. Appl Environ Microbiol 83:e00132-17. doi:10.1128/AEM.00132-17 28389535 PMC5452824

[49] Li F, Li X, Cheng CC, Bujdoš D, Tollenaar S, Simpson DJ, Tasseva G, Perez-Muñoz ME, Frese S, Gänzle MG, Walter J, Zheng J. 2023. A phylogenomic analysis of Limosilactobacillus reuteri reveals ancient and stable evolutionary relationships with rodents and birds and zoonotic transmission to humans. BMC Biol 21:53. doi:10.1186/s12915-023-01541-1 36907868 PMC10010030

[50] Oh PL, Benson AK, Peterson DA, Patil PB, Moriyama EN, Roos S, Walter J. 2010. Diversification of the gut symbiont Lactobacillus reuteri as a result of host-driven evolution. ISME J 4:377–387. doi:10.1038/ismej.2009.123 19924154

[51] Frese SA, Benson AK, Tannock GW, Loach DM, Kim J, Zhang M, Oh PL, Heng NCK, Patil PB, Juge N, Mackenzie DA, Pearson BM, Lapidus A, Dalin E, Tice H, Goltsman E, Land M, Hauser L, Ivanova N, Kyrpides NC, Walter J. 2011. The evolution of host specialization in the vertebrate gut symbiont Lactobacillus reuteri. PLoS Genet 7:e1001314. doi:10.1371/journal.pgen.1001314 21379339 PMC3040671

[52] Lin XB, Wang T, Stothard P, Corander J, Wang J, Baines JF, Knowles SCL, Baltrūnaitė L, Tasseva G, Schmaltz R, Tollenaar S, Cody LA, Grenier T, Wu W, Ramer-Tait AE, Walter J. 2018. The evolution of ecological facilitation within mixed-species biofilms in the mouse gastrointestinal tract. ISME J 12:2770–2784. doi:10.1038/s41396-018-0211-0 30013162 PMC6193996

[53] Tannock GW, Wilson CM, Loach D, Cook GM, Eason J, O’Toole PW, Holtrop G, Lawley B. 2012. Resource partitioning in relation to cohabitation of Lactobacillus species in the mouse forestomach. ISME J 6:927–938. doi:10.1038/ismej.2011.161 22094343 PMC3329185

[54] Shi WW, Jiang YL, Zhu F, Yang YH, Shao QY, Yang HB, Ren YM, Wu H, Chen YX, Zhou CZ. 2014. Structure of a novel O-linked N-acetyl-D-glucosamine (O-GlcNAc) transferase, GtfA, reveals insights into the glycosylation of pneumococcal serine-rich repeat adhesins. J Biol Chem 289:20898–20907. doi:10.1074/jbc.M114.581934 24936067 PMC4110296

[55] Takamatsu D, Bensing BA, Sullam PM. 2004. Genes in the accessory sec locus of Streptococcus gordonii have three functionally distinct effects on the expression of the platelet-binding protein GspB. Mol Microbiol 52:189–203. doi:10.1111/j.1365-2958.2004.03978.x 15049820

[56] Lizcano A, Sanchez CJ, Orihuela CJ. 2012. A role for glycosylated serine-rich repeat proteins in gram-positive bacterial pathogenesis. Mol Oral Microbiol 27:257–269. doi:10.1111/j.2041-1014.2012.00653.x 22759311 PMC3390760

[57] Frese SA, Mackenzie DA, Peterson DA, Schmaltz R, Fangman T, Zhou Y, Zhang C, Benson AK, Cody LA, Mulholland F, Juge N, Walter J. 2013. Molecular characterization of host-specific biofilm formation in a vertebrate gut symbiont. PLoS Genet 9:e1004057. doi:10.1371/journal.pgen.1004057 24385934 PMC3873254

[58] Lee JY, Han GG, Kim EB, Choi YJ. 2017. Comparative genomics of Lactobacillus salivarius strains focusing on their host adaptation. Microbiol Res 205:48–58. doi:10.1016/j.micres.2017.08.008 28942844

[59] Boekhorst J, Helmer Q, Kleerebezem M, Siezen RJ. 2006. Comparative analysis of proteins with a mucus-binding domain found exclusively in lactic acid bacteria. Microbiology (Reading) 152:273–280. doi:10.1099/mic.0.28415-0 16385136

[60] MacKenzie DA, Jeffers F, Parker ML, Vibert-Vallet A, Bongaerts RJ, Roos S, Walter J, Juge N. 2010. Strain-specific diversity of mucus-binding proteins in the adhesion and aggregation properties of Lactobacillus reuteri. Microbiology (Reading) 156:3368–3378. doi:10.1099/mic.0.043265-0 20847011

[61] Yu J, Zhao J, Song YQ, Zhang JC, Yu ZJ, Zhang HP, Sun ZH. 2018. Comparative genomics of the herbivore gut symbiont Lactobacillus reuteri reveals genetic diversity and lifestyle adaptation. Front Microbiol 9:1151. doi:10.3389/fmicb.2018.01151 29915568 PMC5994480

[62] Whelan F, Lafita A, Griffiths SC, Cooper REM, Whittingham JL, Turkenburg JP, Manfield IW, St. John AN, Paci E, Bateman A, Potts JR. 2019. Defining the remarkable structural malleability of a bacterial surface protein Rib domain implicated in infection. Proc Natl Acad Sci USA 116:26540–26548. doi:10.1073/pnas.1911776116 31818940 PMC6936399

[63] Konings WN, Lolkema JS, Bolhuis H, van Veen HW, Poolman B, Driessen AJM. 1997. The role of transport processes in survival of lactic acid bacteria - energy transduction and multidrug resistance. Antonie Van Leeuwenhoek 71:117–128. doi:10.1023/A:1000143525601 9049023

[64] Molenaar D, Bosscher JS, ten Brink B, Driessen AJ, Konings WN. 1993. Generation of a proton motive force by histidine decarboxylation and electrogenic histidine/histamine antiport in Lactobacillus buchneri. J Bacteriol 175:2864–2870. doi:10.1128/jb.175.10.2864-2870.1993 8387991 PMC204603

[65] Wolken WAM, Lucas PM, Lonvaud-Funel A, Lolkema JS. 2006. The mechanism of the tyrosine transporter TyrP supports a proton motive tyrosine decarboxylation pathway in Lactobacillus brevis. J Bacteriol 188:2198–2206. doi:10.1128/JB.188.6.2198-2206.2006 16513749 PMC1428153

[66] Pereira CI, Matos D, San Romão MV, Crespo MTB. 2009. Dual role for the tyrosine decarboxylation pathway in Enterococcus faecium E17: response to an acid challenge and generation of a proton motive force. Appl Environ Microbiol 75:345–352. doi:10.1128/AEM.01958-08 19011061 PMC2620722

[67] Diaz M, Del Rio B, Ladero V, Redruello B, Fernández M, Martin MC, Alvarez MA. 2020. Histamine production in Lactobacillus vaginalis improves cell survival at low pH by counteracting the acidification of the cytosol. Int J Food Microbiol 321:108548. doi:10.1016/j.ijfoodmicro.2020.108548 32050139

[68] Su MS, Schlicht S, Gänzle MG. 2011. Contribution of glutamate decarboxylase in Lactobacillus reuteri to acid resistance and persistence in sourdough fermentation. Microb Cell Fact 10:S8. doi:10.1186/1475-2859-10-S1-S8 21995488 PMC3231934

[69] Ravindran V. 2013. Feed enzymes: the science, practice, and metabolic realities. J Appl Poultry Res 22:628–636. doi:10.3382/japr.2013-00739

[70] Donnet-Hughes A, Rochat F, Serrant P, Aeschlimann JM, Schiffrin EJ. 1999. Modulation of nonspecific mechanisms of defense by lactic acid bacteria: effective dose. J Dairy Sci 82:863–869. doi:10.3168/jds.S0022-0302(99)75304-X 10342225

[71] Davoren MJ, Liu J, Castellanos J, Rodríguez-Malavé NI, Schiestl RH. 2019. A novel probiotic, Lactobacillus johnsonii 456, resists acid and can persist in the human gut beyond the initial ingestion period. Gut Microbes 10:458–480. doi:10.1080/19490976.2018.1547612 30580660 PMC6748577

[72] Denou E, Pridmore RD, Berger B, Panoff JM, Arigoni F, Brüssow H. 2008. Identification of genes associated with the long-gut-persistence phenotype of the probiotic Lactobacillus johnsonii strain NCC533 using a combination of genomics and transcriptome analysis. J Bacteriol 190:3161–3168. doi:10.1128/JB.01637-07 18223069 PMC2347406

[73] Ibnou-Zekri N, Blum S, Schiffrin EJ, von der Weid T. 2003. Divergent patterns of colonization and immune response elicited from two intestinal Lactobacillus strains that display similar properties in vitro. Infect Immun 71:428–436. doi:10.1128/IAI.71.1.428-436.2003 12496193 PMC143181

[74] Seung-Hun L, Eun-Hee Y, Hyuk-Sang K, Jae-Hoon K. 2008. Potential probiotic properties of Lactobacillus johnsonii IDCC 9203 isolated from infant feces. Microbiol Biotechnol Lett 36:121–127.

[75] Lee MD. 2019. GToTree: a user-friendly workflow for phylogenomics. Bioinformatics 35:4162–4164. doi:10.1093/bioinformatics/btz188 30865266 PMC6792077

[76] Aramaki T, Blanc-Mathieu R, Endo H, Ohkubo K, Kanehisa M, Goto S, Ogata H. 2020. KofamKOALA: KEGG ortholog assignment based on profile HMM and adaptive score threshold. Bioinformatics 36:2251–2252. doi:10.1093/bioinformatics/btz859 31742321 PMC7141845

[77] Zhao Y, Jia X, Yang J, Ling Y, Zhang Z, Yu J, Wu J, Xiao J. 2014. PanGP: a tool for quickly analyzing bacterial pan-genome profile. Bioinformatics 30:1297–1299. doi:10.1093/bioinformatics/btu017 24420766 PMC3998138

[78] Snipen L, Liland KH. 2015. Micropan: an R-package for microbial pan-genomics. BMC Bioinformatics 16:79. doi:10.1186/s12859-015-0517-0 25888166 PMC4375852

[79] Shaiber A, Willis AD, Delmont TO, Roux S, Chen L-X, Schmid AC, Yousef M, Watson AR, Lolans K, Esen ÖC, Lee STM, Downey N, Morrison HG, Dewhirst FE, Mark Welch JL, Eren AM. 2020. Functional and genetic markers of niche partitioning among enigmatic members of the human oral microbiome. Genome Biol 21:292. doi:10.1186/s13059-020-02195-w 33323122 PMC7739484

[80] Lopez-Medina E, Neubauer MM, Pier GB, Koh AY. 2011. RNA isolation of Pseudomonas aeruginosa colonizing the murine gastrointestinal tract. J Vis Exp, no. 55:3293. doi:10.3791/3293 21989513 PMC3230207

[81] Ravi K, Falkowski NR, Scales BS, Akulava VD, Valentovich LN, Huffnagle GB. 2022. The psychrotrophic Pseudomonas lundensis, a non-aeruginosa pseudomonad, has a type III secretion system of the ysc family, which is transcriptionally active at 37°C. mBio 13:e03869-21. doi:10.1128/mbio.03869-21 35189702 PMC8903896

