# Supplementary material for: Genomic and transcriptomic insights into vertebrate host-specific Lactobacillus johnsonii adaptation in the gastrointestinal tract
Source: mSphere. 2025 May 13;10(6):e00052-25. doi: 10.1128/msphere.00052-25 (PMC12188725; doi:10.1128/msphere.00052-25)
Supplement: Supplemental information — Supplemental text, figures, and table captions. [file msphere.00052-25-s0001.docx]

**Supplemental methods:**

**Data selection**

The genome assembly of 55 *L. johnsonii* isolates was initially downloaded from the NCBI RefSeq database. These isolates were selected based on isolation source, and various niches of GI tract from vertebrate hosts. For comparative analysis, it was essential to have enough strain representing each host. For this reason, 4 strains, isolated from macaque, cattle, and horse, were removed due to an insufficient number of representative strains for the host. Additionally, to prevent sampling bias in the analysis, strains submitted under the same BioProject ID were further filtered. A representative strain was selected from such BioProject submissions, by setting the following thresholds: (1) pairwise whole genome sequence average nucleotide identity (ANI) score of <0.999 (2) the least number of sequences contigs in the group, and (3) the largest relative length of longest and shortest contig. This collectively led to a final dataset of 42 vertebrate GI tract genome sequences (Table S1).

**Genome annotation**

The genome assemblies of 42 *L johnsonii* strains were downloaded in fasta format. All downloaded files were either complete genomes, with or without plasmid sequence, or had an average of 26 contigs. A closely related species, *L. gasseri* (BIO6369) was used as the outgroup. The headers of the fasta files were first reformed using anvi-script-reformat-fasta. Each fasta file was then converted into a database using anvi-gen-contigs-database. As several gene assemblies had over 30 contigs, any contigs that were less than 2500bp were removed from the database Open reading frames (ORFs) for each database were identified using Prodigal (1). Identified gene calls were annotated by running anvi-run-hmms (2) on the contig database, and functions were assigned by blasting the sequence against the NCBI COGS database using annotated done using anvi-run-ncbi. Functional annotations were also assigned by running anvi-run-kegg-kofams, which uses an Hidden Markov-Model (hmm) search to find hits from the KEGG KOfam database (3). Gene taxonomy was annotated using the Genome Taxonomy Database (GTDB) and anvi-run-scg-taxonomy (4). tRNA sequences were identified using anvi-scan-trnas which uses tRNAScan-SE (5). Following annotations, a summary of the contig databases was then generated using anvi-display-contigs-stats.

**Average nucleotide identity analysis**

For the phylogenomic analysis, *L. gasseri* BIO6369 was selected as the outgroup. A contig database was generated for BIO6369 as described in the previous section. A whole genome-based average nucleotide identity analysis was then run using anvi-compute-genome-similarity, setting the program parameter to pyANI (for < 50 genome sequence) or fastANI (for > 50 genome sequences) (6). The ANIb_percentage_identity text file generated was then imported into R and visualized using ComplexHeatmap. The rows and columns of the heatmap were clustered based on Ward.D2 method of hierarchical clustering. The ANIb percentage identity score for 42 *L. johnsonii* strains was also visualized as a histogram using the ggplot in R.

**Pangenome and Core Genome Analysis**

The pangenome of all 42 *L. johnsonii* genomes was computed using ‘anvi-pan-genome’ with –mcl-inﬂation 10 and --use-ncbi-blast, to cluster the annotated GCs into groups. A similar pangenome was built along with *L. gaeserii* BIO6369 as the out-group. SCGs phylogenetic analysis as done following the SCGs were extracted from this pangenome using anvi-get-sequences-for-gene-clusters with --max-num-genes-from-each-genome 1, --min-num-genomes-gene-cluster- occurs 43 and --min-geometric-homogeneity-index 1. The extracted sequences were cleaned up by removing nucleotide positions that were gap characters in more than 50% of the sequences using trimAl(7). Phylogenetic analysis was done using iqtree with ‘WAG’ general matrix model and 1000 bootstrap(8). The maximum likelihood tree generated was visualized using phylo.io (9).

The anvio summary data, generated using anvi-summarize, was then imported into R, and converted into a pan matrix. The pan matrix was imported into PanGP to visualize the pangenome and core genome profile generated using a totally random (TR) sampling algorithm, with sample size at 500 and sample repeat at 100 (10). The pan matrix data structure was also used with R package micropan to generate Healps’s law alpha value and Jaccard distance matrix (11). Correlation between the subclades, and source of the strains was analyzed using lm() function in R.

**Functional enrichment analysis and identification of rodent-specific genes in *L. johnsonii***

Using both the COG and KEGG annotation, clade-speciﬁc, subclade, and host-specific functions were identiﬁed by the ‘anvi-compute-functional-enrichment-in-pan(12). Functions were considered significantly enriched if they had <0.05 adjusted q-value score, which represents the false-discovery rate adjusted p-value corrected for multiple testing, and >20 enrichment score. Rodent unique GC’s were identified by filtering the anvi-summarize data, selecting for GCs that are present only in rodent isolates, particularly in MR1 using an R script available on [Github](https://github.com/krthkkrv/Genomic-and-transcriptomic-analysis-of-vertebrate-host-specific-lactobacillus-johnsonii).

1. Hyatt D, Chen GL, Locascio PF, Land ML, Larimer FW, Hauser LJ. 2010. Prodigal: prokaryotic gene recognition and translation initiation site identification. BMC Bioinformatics 11:119.

2. Lee MD. 2019. GToTree: a user-friendly workflow for phylogenomics. Bioinformatics 35:4162-4164.

3. Aramaki T, Blanc-Mathieu R, Endo H, Ohkubo K, Kanehisa M, Goto S, Ogata H. 2020. KofamKOALA: KEGG Ortholog assignment based on profile HMM and adaptive score threshold. Bioinformatics 36:2251-2252.

4. Parks DH, Chuvochina M, Waite DW, Rinke C, Skarshewski A, Chaumeil PA, Hugenholtz P. 2018. A standardized bacterial taxonomy based on genome phylogeny substantially revises the tree of life. Nat Biotechnol 36:996-1004.

5. Chan PP, Lowe TM. 2019. tRNAscan-SE: Searching for tRNA Genes in Genomic Sequences. Methods Mol Biol 1962:1-14.

6. Pritchard L, Glover RH, Humphris S, Elphinstone JG, Toth IK. 2016. Genomics and taxonomy in diagnostics for food security: soft-rotting enterobacterial plant pathogens. Analytical Methods 8:12-24.

7. Capella-Gutierrez S, Silla-Martinez JM, Gabaldon T. 2009. trimAl: a tool for automated alignment trimming in large-scale phylogenetic analyses. Bioinformatics 25:1972-3.

8. Nguyen LT, Schmidt HA, von Haeseler A, Minh BQ. 2015. IQ-TREE: a fast and effective stochastic algorithm for estimating maximum-likelihood phylogenies. Mol Biol Evol 32:268-74.

9. Robinson O, Dylus D, Dessimoz C. 2016. Phylo.io: Interactive viewing and comparison of large phylogenetic trees on the web. Mol Biol Evol 33:2163-6.

10. Zhao Y, Jia X, Yang J, Ling Y, Zhang Z, Yu J, Wu J, Xiao J. 2014. PanGP: a tool for quickly analyzing bacterial pan-genome profile. Bioinformatics 30:1297-9.

11. Snipen L, Liland KH. 2015. micropan: an R-package for microbial pan-genomics. BMC Bioinformatics 16:79.

12. Shaiber A, Willis AD, Delmont TO, Roux S, Chen LX, Schmid AC, Yousef M, Watson AR, Lolans K, Esen ÖC, Lee STM, Downey N, Morrison HG, Dewhirst FE, Welch JLM, Eren AM. 2020. Functional and genetic markers of niche partitioning among enigmatic members of the human oral microbiome. Genome Biology 21.

**Supplemental figures**


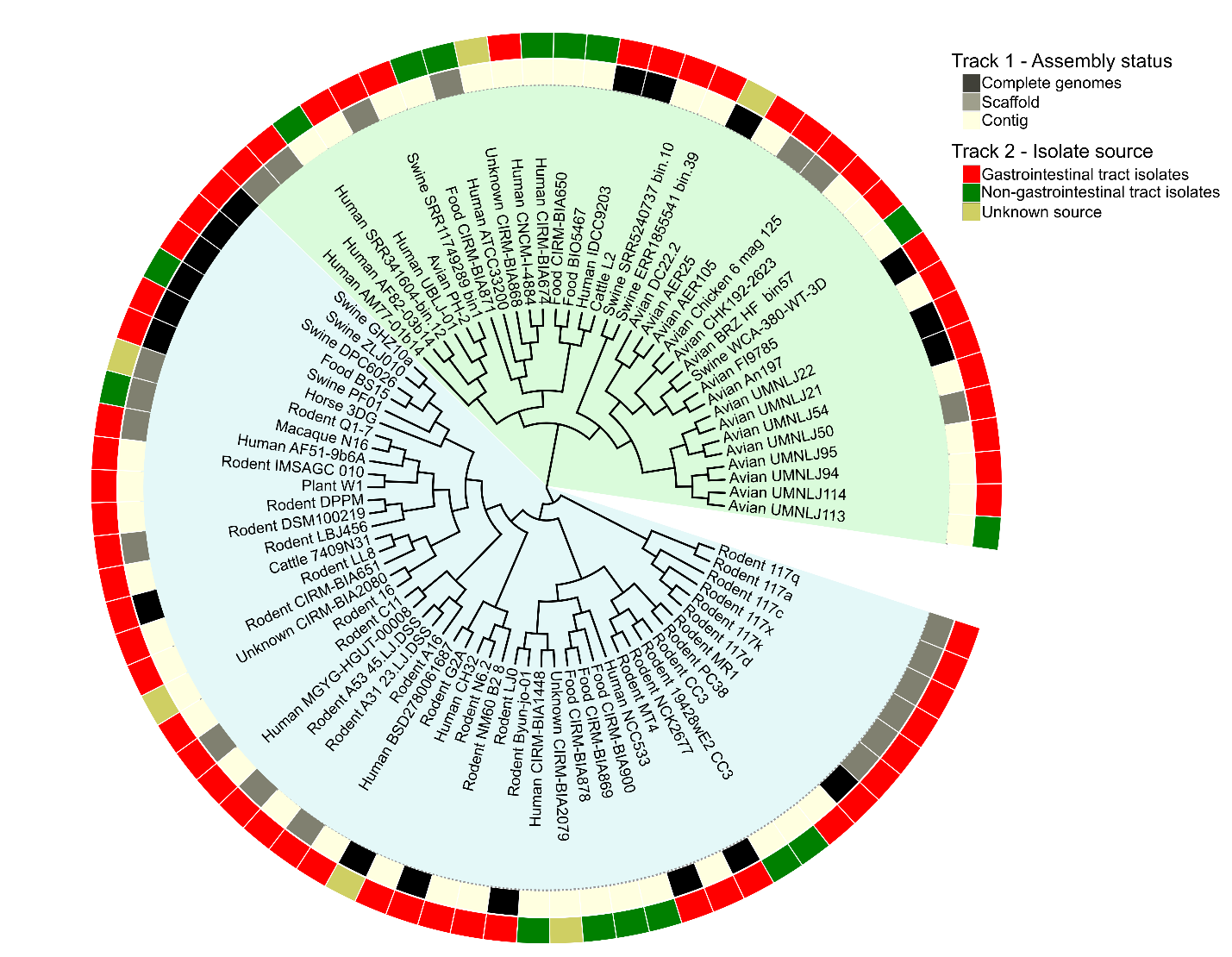


**Fig S1. Phylogenetic clustering of *L. johnsonii* strains based on whole genome sequence ANI scores.** All the strains in the analysis are split into two clades, one clade containing all the avian isolates and the other containing all the rodent isolates in the study. Non-GI tract isolates were distributed among these two clades and did not form a distinct clade of its own. The inner annotation circle represents the assembly status of the strains; black, complete genome sequence; gray, Scaffolds and light yellow, contigs. The outer layer represents the source of the isolates; red, GI tract isolates; green, non-GI tract isolates, including food isolates and dark yellow, isolates of unknown source.


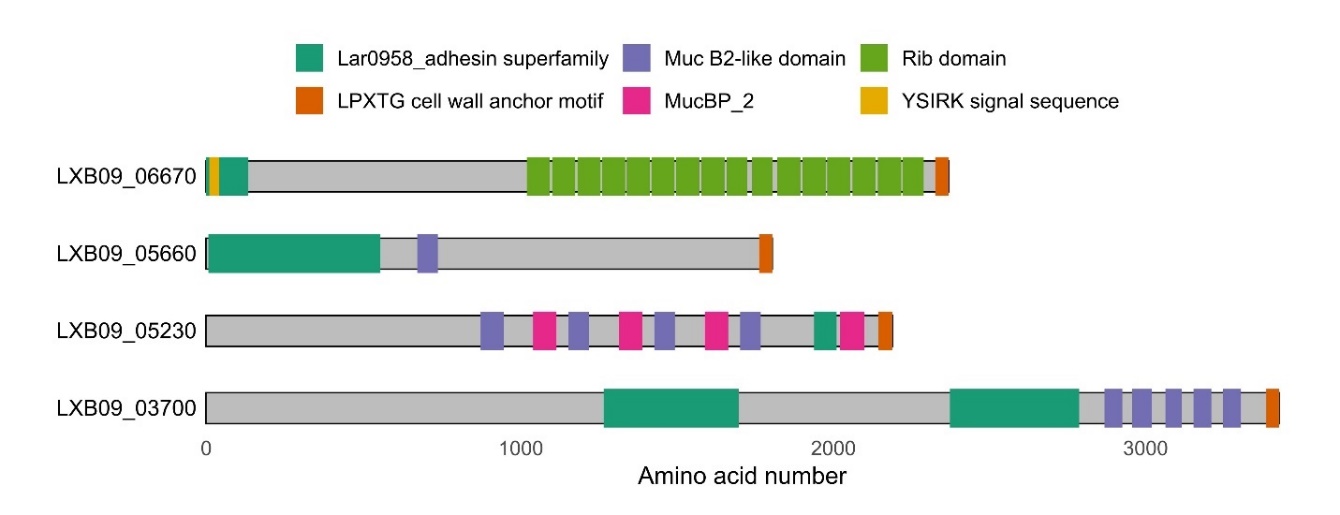


**Fig S2. Conserved domains in rodent-specific Surface proteins identified in L. johnsonii MR1.** Four putative surface proteins were identified as significantly enriched among rodent isolates, highlighting the conserved domains and motifs associated with adhesion, mucus binding, cell wall anchor, and protein export signal.


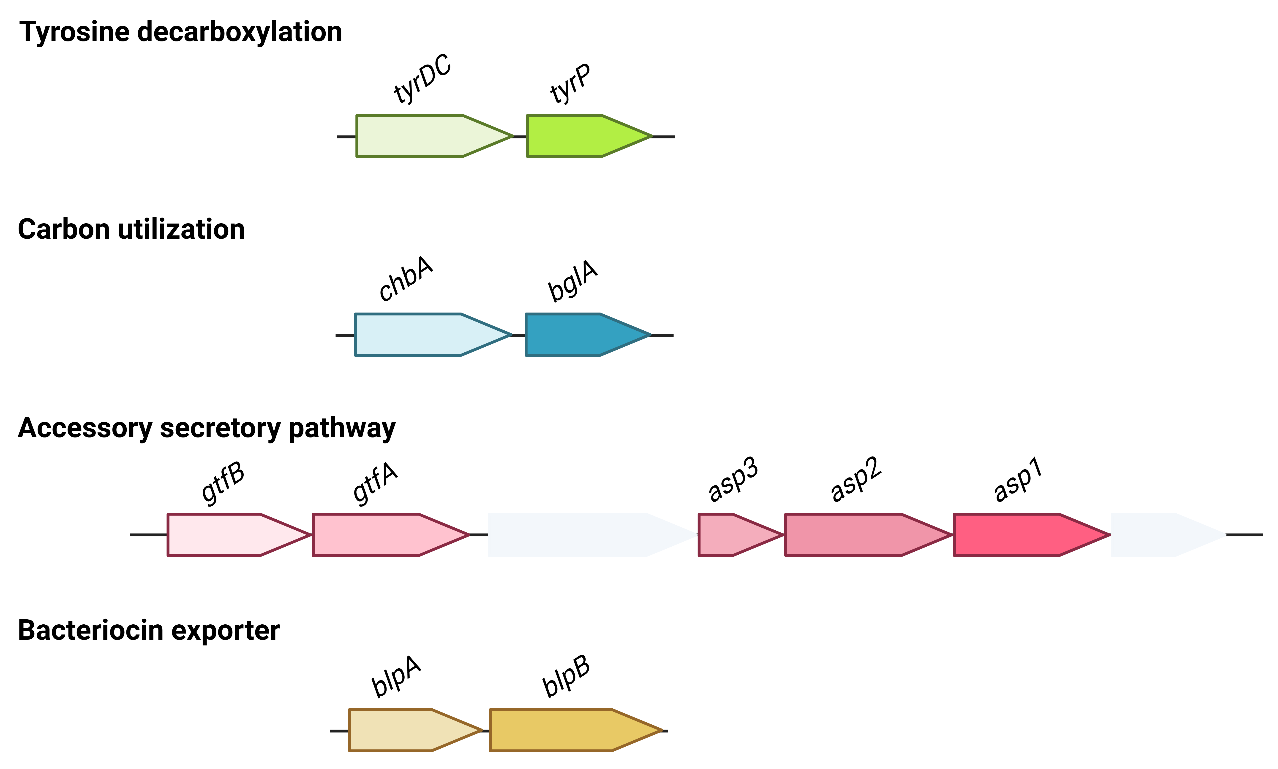


**Fig S3. Genes in MR1 are identified as enriched among rodent isolates or Unique to rodent isolates.** Genes associated with tyrosine decarboxylation and carbon utilization are identified as unique to rodent isolates and absent in strains from other hosts. Accessory secretory pathway and bacteriocin exporter-related genes are significantly enriched among rodent isolates and are absent in the avian isolates in the study. Created with BioRender.com.


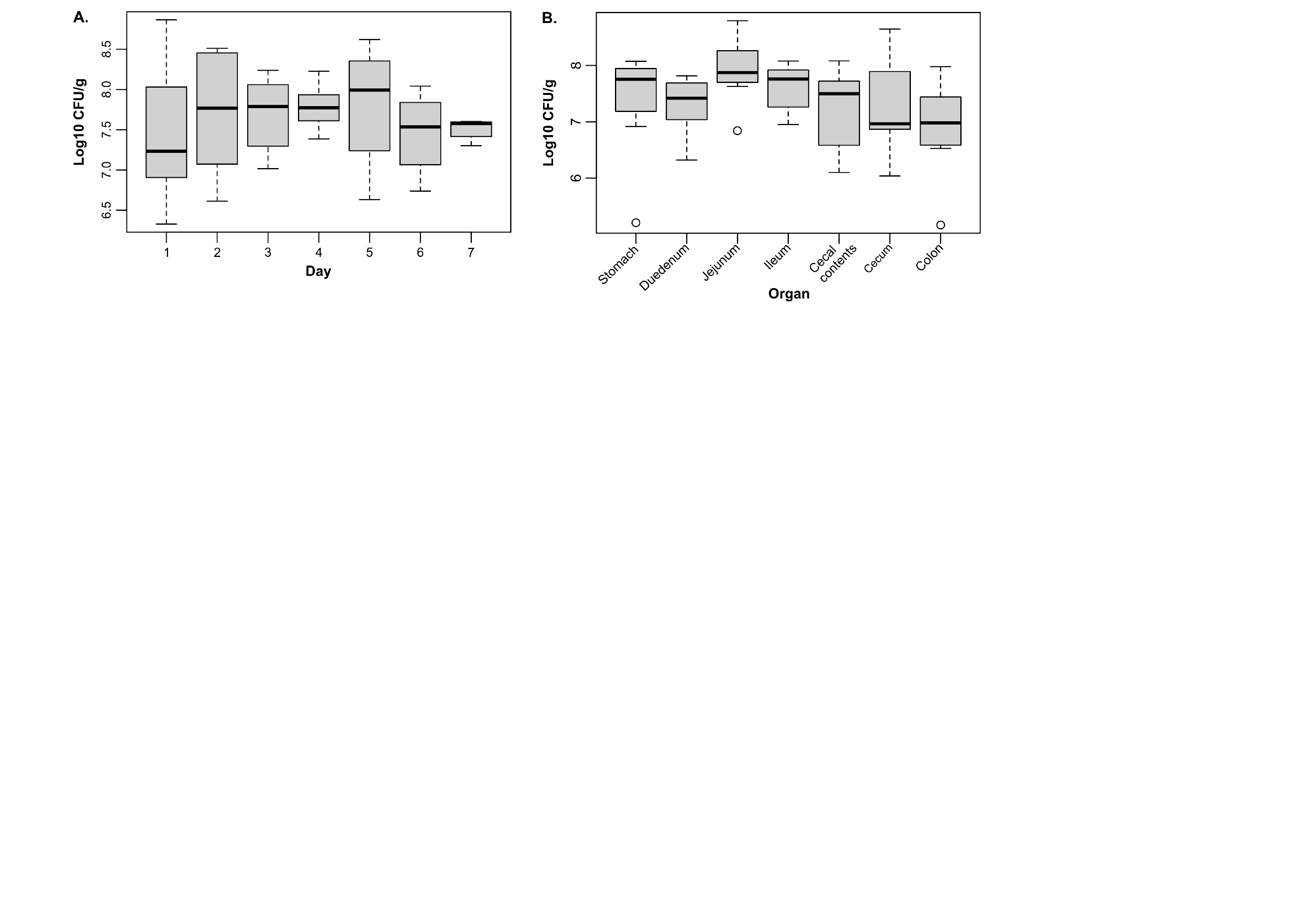
**Fig S4. *L. johnsonii* MR1 persistence in the gut of germ-free mice.** CFU levels of *L. johnsonii* MR1 in feces of germ-free Balb/c mice (n = 8) orally gavaged with 3x10^8^ CFU of *L. johnsonii* MR1 on day 0 (A). CFU/g levels of *L. johnsonii MR1* in the various niches of the GI tract, on the 7th day after oral gavage of Balb/c mice.


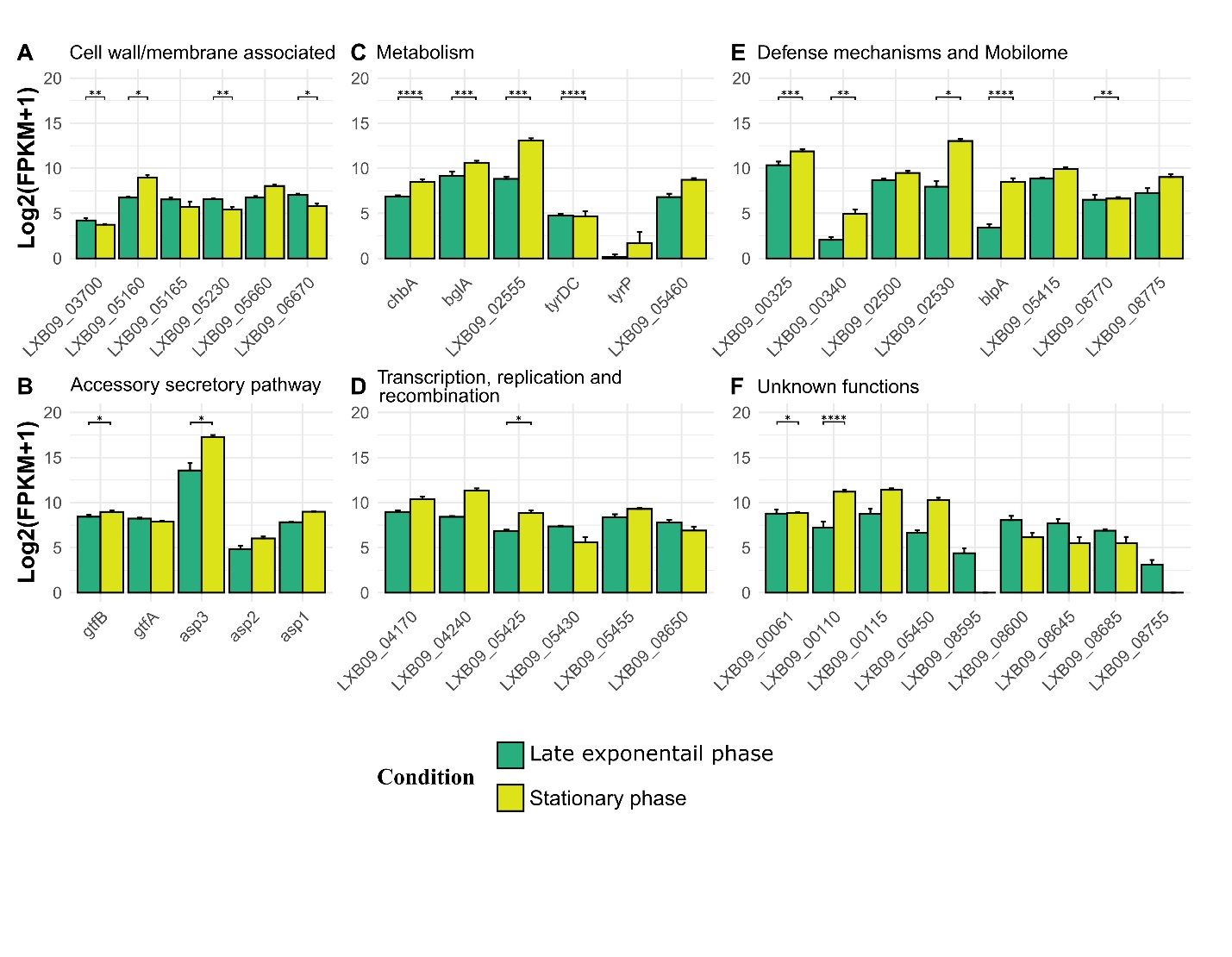


**Fig S5. Anaerobic late exponential phase vs stationary phase expression of rodent-specific genes in *L. johnsonii* MR1.** Bar plot representing Fragments Per Kilobase of transcript per Million mapped (FPKM) reads of *L. johnsonii* during the anaerobic late exponential growth phase (green bars) and anaerobic stationary growth phase (yellow bars). Genes are binned into groups based on their functional annotations. While all the rodent-specific genes were expressed during *in vitro* growth, several genes were significantly differentially regulated. Notably, *tyrP* while expressed during the stationary phase is transcriptionally inactive during the late exponential phase. Similarly, LXB09_08595 and _08755 are only expressed during the late exponential phase an not the stationary phase. The asterisk located above the black line indicates genes with significant changes in expression between the two conditions. One asterisk (*) indicates an adjusted p-value smaller than 0.05 (p<0.05), two asterisks (**) indicate an adjusted p-value smaller than 0.01 (<0.01) three asterisks (***) indicate an adjusted p-value smaller than 0.001 (p<0.001).

**Supplemental tables**

**Table S1:** 42 *L. johnsonii* strains used in the comparative genomic analysis. These strains were reported to be isolated from various niches of the GI tract, including fecal samples.

**Table S2:** Pangenome summary of *42 L. johnsonii* vertebrate GI tract isolates, built using Anvi'o microbial pangenomics analysis pipeline.

**Table S3:** Functional enrichment analysis across strains in 4 host groups.

**Table S4:** Pairwise whole genome sequence average nucleotide identity score for 42 *L. johnsonii* strains and the outgroup *L. gasseri* BIO6369.

**Table S5:** Log fold change in expression of genes in *L. johnsonii* MR1 during *in vitro* and *in vivo* growth.

**Table S6:** Log fold change in expression of the 40 rodent-associated genes in *L. johnsonii* MR1 during *in vitro* and *in vivo* growth.
